# Association between cerebrospinal fluid clusterin and biomarkers of Alzheimer’s disease pathology in mild cognitive impairment: a longitudinal cohort study

**DOI:** 10.3389/fnagi.2023.1256389

**Published:** 2023-10-24

**Authors:** Hao Wang, Ling-Zhi Ma, Ze-Hu Sheng, Jia-Yao Liu, Wei-Yu Yuan, Fan Guo, Wei Zhang, Lan Tan

**Affiliations:** ^1^Department of Neurology, Affiliated Hospital of Weifang Medical University, School of Clinical Medicine, Weifang Medical University, Weifang, China; ^2^Department of Neurology, Qingdao Municipal Hospital, Qingdao University, Qingdao, China; ^3^Department of Neurology, Qingdao Municipal Hospital, University of Health and Rehabilitation Sciences, Qingdao, China

**Keywords:** Alzheimer’s disease, cerebrospinal fluid, biomarkers, cognition, clusterin

## Abstract

**Background:**

Clusterin, a glycoprotein implicated in Alzheimer’s disease (AD), remains unclear. The objective of this study was to analyze the effect of cerebrospinal fluid (CSF) clusterin in relation to AD biomarkers using a longitudinal cohort of non-demented individuals.

**Methods:**

We gathered a sample comprising 86 individuals under cognition normal (CN) and 134 patients diagnosed with MCI via the Alzheimer’s Disease Neuroimaging Initiative (ADNI) database. To investigate the correlation of CSF clusterin with cognitive function and markers of key physiological changes, we employed multiple linear regression and mixed-effect models. We undertook a causal mediation analysis to inspect the mediating influence of CSF clusterin on cognitive abilities.

**Results:**

Pathological characteristics associated with baseline Aβ_42_, Tau, brain volume, exhibited a correlation with initial CSF clusterin in the general population, Specifically, these correlations were especially prominent in the MCI population; CSF Aβ_42_ (P_CN_ = 0.001; P_MCI_ = 0.007), T-tau (P_CN_ < 0.001; P_MCI_ < 0.001), and Mid temporal (P_CN_ = 0.033; P_MCI_ = 0.005). Baseline CSF clusterin level was predictive of measurable cognitive shifts in the MCI population, as indicated by MMSE (*β* = 0.202, *p* = 0.029), MEM (*β* = 0.186, *p* = 0.036), RAVLT immediate recall (*β* = 0.182, *p* = 0.038), and EF scores (*β* = 0.221, *p* = 0.013). In MCI population, the alterations in brain regions (17.87% of the total effect) mediated the effect of clusterin on cognition. It was found that variables such as age, gender, and presence of *APOE ε4* carrier status, influenced some of these connections.

**Conclusion:**

Our investigation underscored a correlation between CSF clusterin concentrations and pivotal AD indicators, while also highlighting clusterin’s potential role as a protective factor for cognitive abilities in MCI patients.

## 1. Introduction

Dementia, a neurological disorder marked by a steady deterioration of cognitive abilities, impacts memory, thought processes, and behavior. Alzheimer’s disease (AD) predominates as the most frequently diagnosed type of dementia, comprising about 70% of all cases (Duong et al., 2017). The amyloid cascade theory suggests that the accumulation of amyloid-beta (Aβ) peptide in cerebral regions marks a critical incident in AD’s progression. This event, occurring quite early, has the potential to set off tau-related pathology, provoking detrimental cellular reactions that may result in neuronal dysfunction and cellular death (Karran et al., 2011; Pooler et al., 2015).

The tau-related pathology observed in neurons aligns closely with the cognitive deterioration typical of the disease’s final stage. There are theories that this process occurs in a continuous cascade (Karran and De Strooper, 2022). Clusterin is a multifunctional glycoprotein encoded by the CLU gene (a single copy gene located on chromosome 8 at the p21-p12 locus), also known as apolipoprotein J (Foster et al., 2019; Yuste-Checa et al., 2022). The protective effect of this protein against cellular stress has been reported in studies of cardiac injury and tumor survival (Djeu and Wei, 2009; Foster et al., 2019). Apart from curtailing the development of amyloid polypeptide fibrils, a key marker of Alzheimer’s pathology, it further mitigates apoptosis and oxidative stress. It exhibits the capability to bind Aβ polypeptides, thus thwarting excessive inflammation (Wu et al., 2012).

One study observed a significant association between single nucleotide polymorphisms (SNPs) at the CLU gene (also known as APOJ) (rs11136000) at a genome-wide level (Harold et al., 2009). It has been reported that clusterin is associated with neuroprotective effects in AD (Wojtas et al., 2020) and has been associated with major AD pathological markers (Tang et al., 2022). Recent researches suggested that the CLU protein may promote the propagation of tau pathology, and an animal study also observed an increase in tau levels in mice treated with CLU (Martin-Rehrmann et al., 2005; Yuste-Checa et al., 2021). The processes through which clusterin influences AD pathology remain ambiguous. The primary focus of our research was to find the correlation between clusterin in CSF, AD pathophysiological processes, and cognitive abilities. Furthermore, we sought to determine the risk factors for AD, such as age, gender, and presence of *APOE ε4* carrier status, influence these correlations (Rosselli et al., 2022).

Additionally, our goal was to determine the degree to which other pertinent biomarkers might influence these correlations. Gaining a comprehensive understanding of how clusterin ties into the onset and clinical presentation of AD could also serve as a beneficial guide for early detection and management of AD. To achieve our goals, we analyzed several biomarkers related to AD pathophysiology such as Aβ_42_, tau protein abnormalities (phosphorylated tau protein, P-tau protein), and indicators of neurodegeneration including total tau protein (T-tau protein) and MRI findings. These biomarkers were chosen for their representation of crucial stages in the AD progression and their extensive research in the context of this disorder (Wang et al., 2020). Our analyses were conducted on a population of non-demented individuals to better understand the early stages of the disease (Giau et al., 2019; Talwar et al., 2021).

## 2. Materials and methods

### 2.1. ADNI database

ADNI is a large-scale research effort launched in 2003, an international multicenter, ongoing, observational object, helps improve our understanding of AD and other disorders of the nervous system through a longitudinal study. Participants undergo regular clinical assessments, neuroimaging scans, and blood and CSF tests. The data collected from participants are stored in a central database and made available to researchers worldwide, see the ADNI website for the latest information https://adni.loni.usc.edu/ (Veitch et al., 2019, 2022).

### 2.2. Participants

Data was retrieved from the ADNI database, encompassing clinical attributes, CSF details, MRI scan results, and cognitive information from 220 participants. The ADNI database classifies subjects as clinically cognitively normal (CN), including those with subjective memory symptoms (MMSE >24, CDR = 0), those with mild cognitive impairment (MCI; MMSE >24, CDR = 0.5), and those with dementia. Participants underwent periodic evaluations for clinical data collection and participation in biomarker studies, which included CSF extraction. The inclusion of 220 individuals necessitated the presence of clusterin protein, demographic information, *APOE* gene carriage status, cognitive diagnosis. Simultaneously, we incorporated cognitive assessment scores of participants meeting these criteria and brain structural organizational data, despite the potential for missing data. Following the baseline examination, biomarker measurements and clinical cognitive assessments were conducted at baseline (BL), 12, 48, 72, 96, 120, 144, 168, and 180 months (Supplementary Table S1). All participants were made aware of the study’s aim and granted their approval via signed consent forms (Aisen et al., 2010).

### 2.3. Measurements of biomarkers

Measurement of Aβ_42_, T-tau, and P-tau in CSF by the INNO-BIA AlzBio3 kit (Shaw et al., 2009). CSF samples were analyzed using an automated Roche Elecsys^®^ platform at the University of Pennsylvania. Further details can be found in previously published literature (Schindler et al., 2018). Repeated biomarker assays in CSF were averaged, and CSF clusterin was quantified using liquid chromatography-tandem mass spectrometry multiple reaction monitoring (LC/MS-MRM) with the peptide sequence IDSLLENDR. The ADNI database was used to collect all observed data (Kennedy et al., 2014; Liu et al., 2022).

### 2.4. MRI assessment

The MRI Core has established a standardized and publicly accessible data analysis set through the implementation of precise and fully automated brain and hippocampal segmentation algorithms, which have been validated in previously published studies. In our study, we focused on the following regions of interest (ROI): the whole brain, hippocampus, entorhinal, fusiform, and middle temporal areas. Previous large cohort studies, such as the Add Neuro Med project in Europe and the ADNI database study, have demonstrated the research value of these brain regions (Mangialasche et al., 2013; Falahati et al., 2017; Yu et al., 2021).

### 2.5. Cognitive assessment

We retrieved the composite memory score (MEM), as well as the commonly used cognitive measures, including ADAS-Cog which full name is the AD Assessment Scale-Cognitive subscale and executive functioning (EF) scores, the Brief Mental State Evaluation Scale (MMSE), and the Rey Auditory Word Learning Immediate Test (RAVLT immediate), from the ADNI Neuropsychological Test to monitor the cognitive measures’ trajectory. All of them are psychometrically optimized composite scores, which have been previously validated and proven to be reliable and externally valid (Folstein et al., 1975; Crane et al., 2012; Gibbons et al., 2012; Xu et al., 2022).

### 2.6. Statistical analysis

CSF clusterin concentrations were analyzed and found to be approximately normally distributed (Supplementary Table S1). To ensure the reliability of the results, We defined outliers as those with a standard deviation of 4 SD above or below the mean, and all analyses in this study were performed using transformed log10 values. We compared continuous demographics, clinical outcomes, and biomarkers using Student’s *t*-test or Wilcoxon rank sum tests, while nominal variables were compared with chi-square test or one-way ANOVA, followed by *post hoc* comparisons. The correlation of baseline CSF clusterin with biomarker and cognitive data was assessed using linear regression models. In each model, CSF clusterin levels were used as the form of independent variables, and dependent variables included cognitive measures and biomarkers. We employed a technique previously adopted by other researchers wherein a Linear Mixed Effects (LME) model was fitted, designating different measures as the dependent variable, The independent variable is time, while adjusting for random slope and intercept (Ma et al., 2021).

We also used the model to extract the rate of change of each variable for further subsequent analysis. We used the main biomarkers mentioned above as our hypothetical mediators to analyze the mediation between CSF clusterin and multiple cognitive performance, all tests are bootstrapped with 10,000 replications and adjusted for covariates. The cumulative risk of cognitive progression during follow-up was compared between groups stratified by clusterin level using Kaplan–Meier curves. Furthermore, the relationship between clusterin and the incidence of cognitive progression during follow-up was analyzed using multivariate Cox regression models. In the present study, we introduce a novel variable derived from the product of AD risk factors and CSF clusterin to the model to evaluate the interactive effects of clusterin levels and known AD risk factors (such as age, gender, and *APOE ε4* status) on the dependent variables. Moreover, we utilized a pre-established and verified cut-off value: a critical concentration of 976.6 pg./mL of CSF Aβ_42_, enabling us to categorize subjects into A+ and A− groups for further analys (Tang et al., 2022).

All statistical analyses were performed in R 0.4.2.2. All regression analyses were corrected for age level, participant gender, educational level, and presence of *APOE ε4* carrier status.

## 3. Results

The main characteristics of the study population, according to cognitive state, are reported in Table 1. We divided the participants into two groups: normal cognitive ability (CN; *n* = 86) and mild cognitive impairment (MCI; *n* = 134).

**Table 1:** Clinical characteristics of participants in individual groups in the current study.

Characteristics	CN (*n* = 86)	MCI (*n* = 134)	*p* value
Age (years)	75.70 ± 5.54	74.69 ± 7.34	0.278
Gender = male (%)	44 (51.2)	91 (67.9)	0.019
Education (years)	15.64 ± 2.97	16.02 ± 2.98	0.353
*APOE ε4* carriers (%)	21 (22.8)	71 (77.2)	<0.001
Aβ_42_	1060.58 ± 386.15	713.68 ± 299.97	<0.001
T-tau	242.35 ± 76.67	313.44 ± 113.11	<0.001
P-tau	22.36 ± 8.10	31.00 ± 12.87	<0.001
Clusterin	20.61 ± 0.44	20.63 ± 0.48	0.712
ADAS11	6.02 ± 2.94	11.49 ± 4.13	<0.001
ADAS13	9.24 ± 4.25	18.91 ± 5.93	<0.001
ADASQ4	2.84 ± 1.79	6.33 ± 2.26	<0.001
MMSE	29.04 ± 1.02	26.99 ± 1.78	<0.001
RAVLT immediate	45.19 ± 8.46	32.04 ± 8.56	<0.001
MEM	0.980 ± 0.50	−0.150 ± 0.57	<0.001
EF	0.600 ± 0.72	−0.110 ± 0.80	<0.001
Ventricles	34480.78 ± 17056.81	43755.98 ± 20122.09	0.001
Hippocampus	7191.51 ± 846.89	6315.79 ± 1099.98	<0.001
Whole brain	997298.94 ± 102276.17	1005024.17 ± 108157.33	0.600
Entorhinal	3792.09 ± 696.59	3305.62 ± 749.94	<0.001
Fusiform	17087.26 ± 2378.97	16643.06 ± 2296.11	0.207
Mid temporal	19588.08 ± 2848.87	18728.40 ± 2901.55	0.049
Dementia at follow up (%)	10 (11.5)	85 (63.9)	<0.001

Categorical variables are reported as numbers and percentages; continuous variables are reported as means ± SDs. Gender, the gender of the participants; Education, Years of education of the participants; CN, cognitively normal; MCI, mild cognitive impairment; APOE, Apolipoprotein E; Aβ_42_, Amyloid-β_42_; T-tau, Total tau; P-tau, Phosphorylated tau; ADAS, Alzheimer’s disease assessment scale-cognitive; ADASQ4, ADAS delayed word recall; MMSE, mini mental state examination; RAVLT immediate, Rey auditory verbal learning test immediate recall; MEM, memory function composite score; EF, executive function composite score.

As expected, cognitive test scores differed significantly between the CN and MCI groups (ADAS11, *p* < 0.001; ADAS13, *p* < 0.001; ADASQ4, *p* < 0.001; MMSE, *p* < 0.001, AVLT immediate, *p* < 0.001; MEM, *p* < 0.001; EF, *p* < 0.001).

Almost all neuroimaging variables, except the whole brain volume and fusiform volume, showed significant differences between the two cognitive groups (ventricles volume, *p* = 0.001; hippocampus volume, *p* < 0.001; entorhinal volume, *p* < 0.001; mid temporal volume, *p* < 0.001). MCI group had higher T-tau (*p* < 0.001), P-tau (*p* < 0.001), and relatively lower levels of CSF Aβ_42_ (*p* < 0.001). There was no clear difference in CSF clusterin and years of education between the two groups which closely resembled previous findings (Tang et al., 2022). The MCI population had a higher proportion of *APOE ε4* carriers compared to the CN group. Additionally, we observed a progressive increase in dementia prevalence from the CN (11.5%) to the MCI group (63.9%), which is by previously reported findings in the literature (Chen et al., 2022).

### 3.1. Cross-sectional CSF biomarker analysis

**Figure 1:**
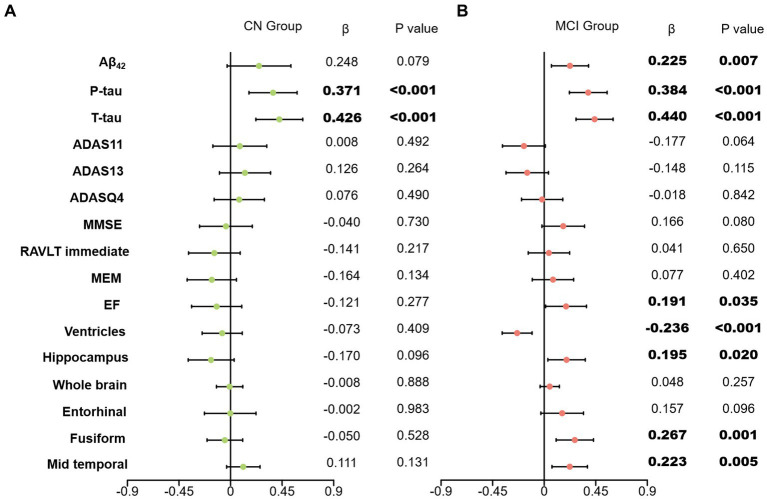
Effects of clusterin with biomarkers and cognitive data. Associations of baseline clusterin on baseline biomarkers and cognitive measurements in two group. All analyses were corrected for age, gender, educational level, *APOE ε4* status, and intracranial volume. **(A)** results of CN group; **(B)** results of MCI group; CN, cognitively normal; MCI, mild cognitive impairment; Aβ_42_, Amyloid-β_42_; T-tau, Total tau; P-tau, Phosphorylated tau; ADAS, Alzheimer’s disease assessment scale-cognitive; ADASQ4, ADAS delayed word recall; MMSE, mini-mental state examination; RAVLT immediate, Rey auditory verbal learning test immediate recall; MEM, memory function composite score; EF, executive function composite score.

**Figure 2:**
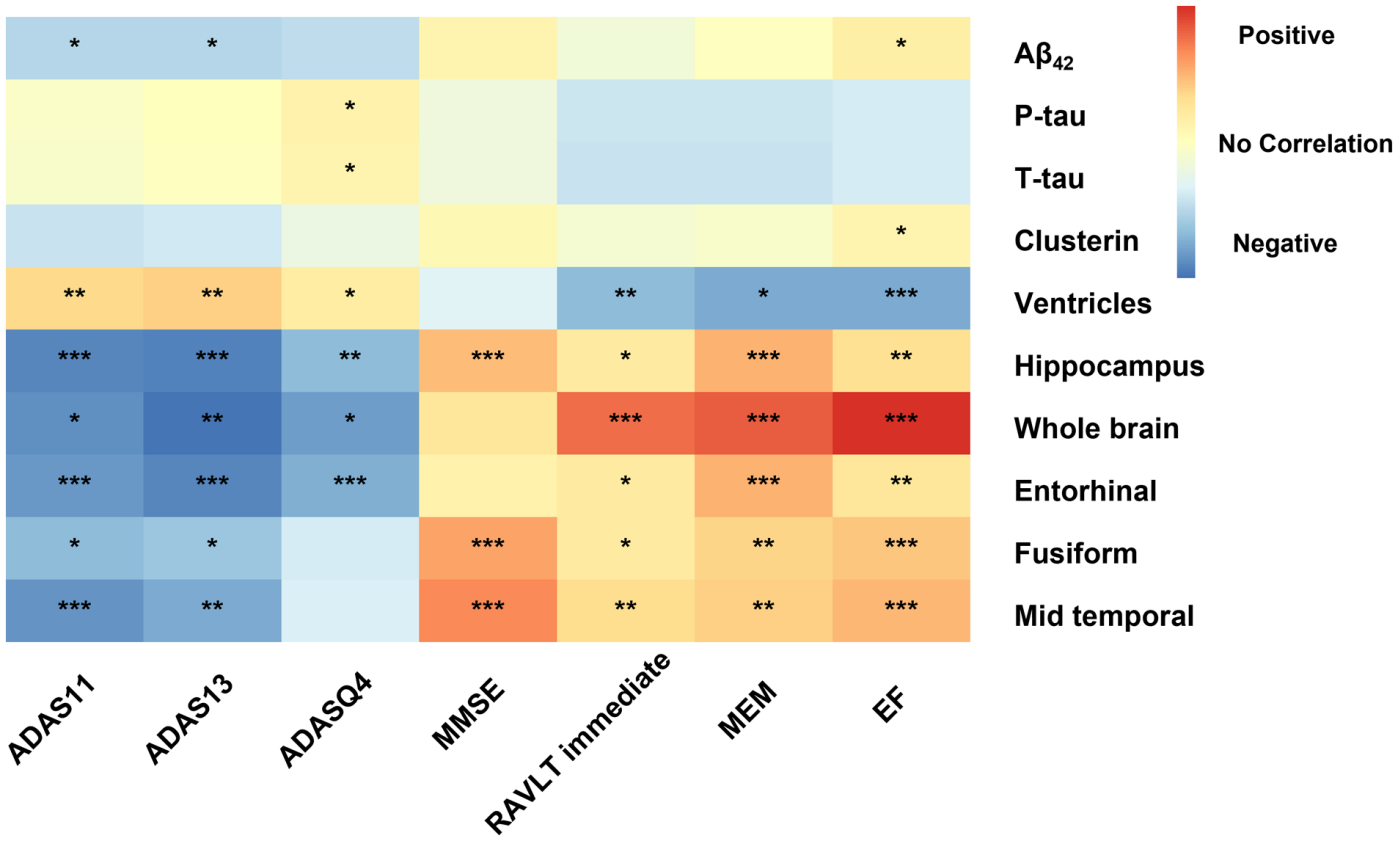
Effects of biomarkers on cognitive measures within MCI participants. Correlation of AD biomarker cognitive measurements at baseline. All analyses were corrected for age, gender, educational level, *APOE ε4* status, and intracranial volume. Significance have been marked with the symbol ***, **, and *: *p* value<0.001 and *p* value <0.01, and *p* value<0.05. Aβ_42_, Amyloid-β_42_; T-tau, Total tau; P-tau, Phosphorylated tau; ADAS, Alzheimer’s disease assessment scale-cognitive; ADASQ4, ADAS delayed word recall; MMSE, mini-mental state examination; RAVLT immediate, Rey auditory verbal learning test immediate recall; MEM, memory function composite score; EF, executive function composite score.

In our primary analysis, we computed the correlations between clusterin and all biomarkers as well as cognitive measures after adjustment of age, gender, educational level, *APOE ε4* carriers’ status, and intracranial volume. A positive correlation was found between clusterin and biomarkers remained significant in the MCI group (CSF Aβ, β = 0.225, *p* = 0.007; Total tau, β = 0.440, *p* < 0.001; P-tau, β = 0.384, *p* < 0.001). EF was found to have a positive correlation with clusterin (β = 0.191, *p* = 0.035), whereas other cognitive factors had no correlation with clusterin. The high level of clusterin correlated with the bigger volumes of the hippocampus (*p* = 0.02), fusiform (*p* = 0.001), mid-temporal (*p* = 0.005), and smaller volumes of ventricles (*p* < 0.001). No significant associations were listed between baseline clusterin and other neuroimaging variables in a cross-sectional study. Further analyses suggested that CSF Aβ, tau, MRI imaging markers, and clusterin directly impact cognitive measurement (Figures 1, 2).

### 3.2. Age, gender, and *APOE ε4* interactions with CSF clusterin on biomarkers

In this analysis, we aimed to examine the interactions between CSF clusterin levels and age, gender, and *APOE ε4* status on various biomarkers in the CN and MCI groups. The main findings were as follows:

Our observations revealed an interaction between CSF clusterin levels and age on T-tau, MRI (hippocampus volume, whole brain volume, and mid-temporal volume), and cognitive measurements (EF) exclusively in MCI participants. In the CN group, the initial interaction effect we examined involved the correlation between CSF clusterin and other measures, with *APOE ε4* status affecting baseline MRI (mid-temporal volume). Interaction effects were noted for longitudinal MRIand cognitive measurements (EF). Interactions are statistically significant between CSF clusterin and gender on longitudinal ADAS11. As for the relationship between age and CSF clusterin, it was noteworthy for longitudinal MRI (ventricles and mid temporal) (Supplementary Tables S8, S9).

### 3.3. Prediction of longitudinal changes of CSF biomarkers, cognitive score, and neuroimaging variables using baseline CSF clusterin

**Figure 3:**
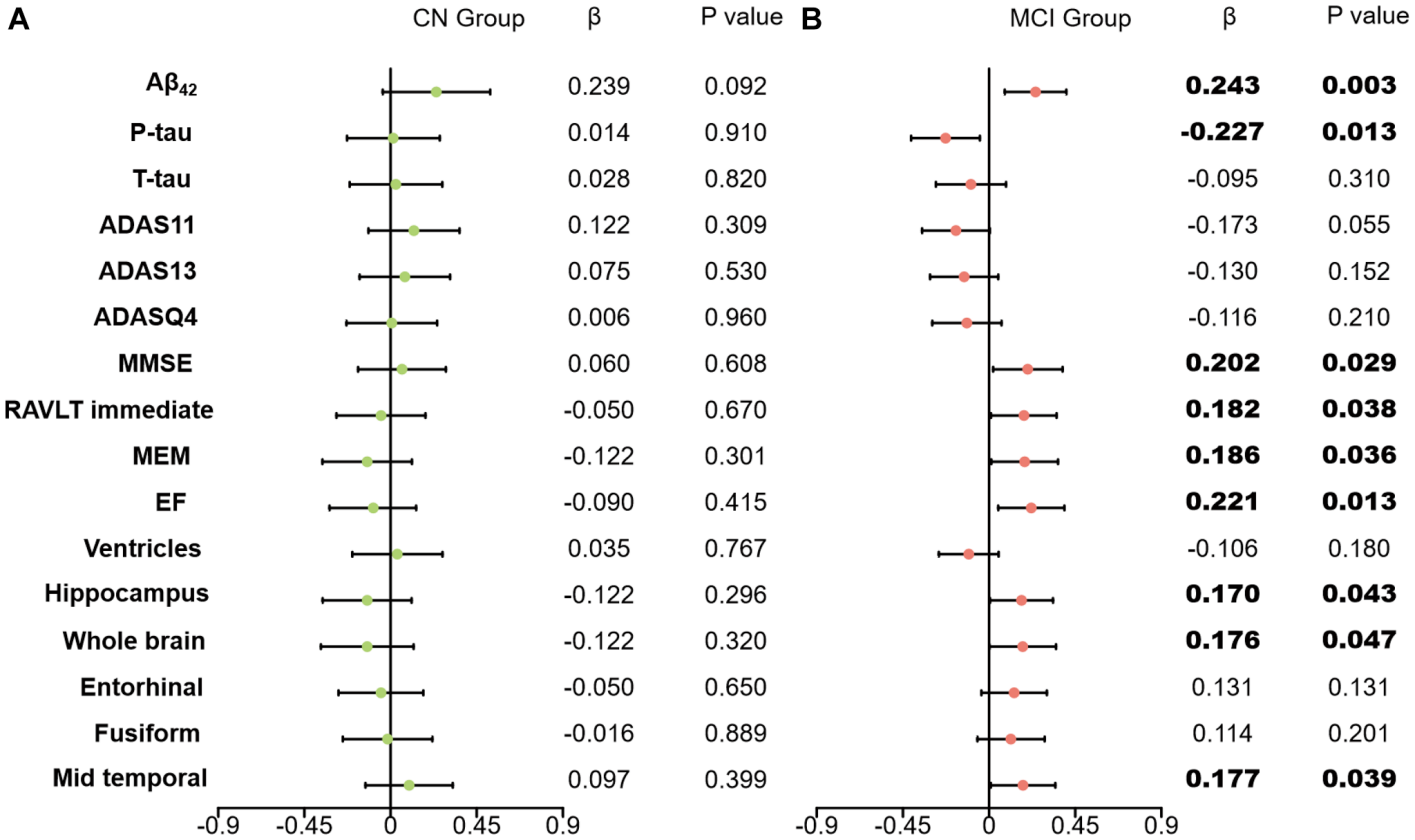
Effects of clusterin on longitudinal biomarkers and cognitive score. All analyses were corrected for age, gender, educational level, *APOE ε4* status, and intracranial volume. **(A)** results of CN group; **(B)** results of MCI group; CN, cognitively normal; MCI, mild cognitive impairment; Aβ_42_, Amyloid-β_42_; T-tau, Total tau; P-tau, Phosphorylated tau; ADAS, Alzheimer’s disease assessment scale-cognitive; ADASQ4, ADAS delayed word recall; MMSE, mini-mental state examination; RAVLT immediate, Rey auditory verbal learning test immediate recall; MEM, memory function composite score; EF, executive function composite score.

In the longitudinal analysis, we examined the relationship between baseline CSF clusterin levels and changes in CSF biomarkers, cognitive scores, and neuroimaging variables over time. We hypothesized that the upregulation of clusterin would be related to an increase in overall cognitive ability. After adjusting for relevant covariates, we found the following results for the MCI group:

A higher baseline CSF clusterin level was significantly associated with a higher increased rate not only of global cognition (MMSE, β = 0.202, *p* = 0.029), composite memory (MEM, β = 0.186, *p* = 0.036) but also of short-term auditory-verbal memory (i.e., RAVLT immediate, β = 0.182, *p* = 0.038), executive function (EF, β = 0.221, *p* = 0.013), Additionally, lower baseline CSF clusterin level predicted greater decline in hippocampus volume (β = 0.170, *p* = 0.043), the whole brain volume (β = 0.176, *p* = 0.047), mid temporal volume (β = 0.177, *p* = 0.039). A higher baseline clusterin level was significantly associated with a higher increased rate of CSF Aβ (β = 0.243, *p* = 0.003). Meanwhile, the upregulation of baseline clusterin predicted a decrease in the rate of change of P-tau (β = −0.227, *p* = 0.013). However the effects of baseline clusterin on longitudinal biomarkers and cognitive were not statistically significant in cognitively normal populations (CN) (Figure 3).

### 3.4. Mediation analyses

**Figure 4:**
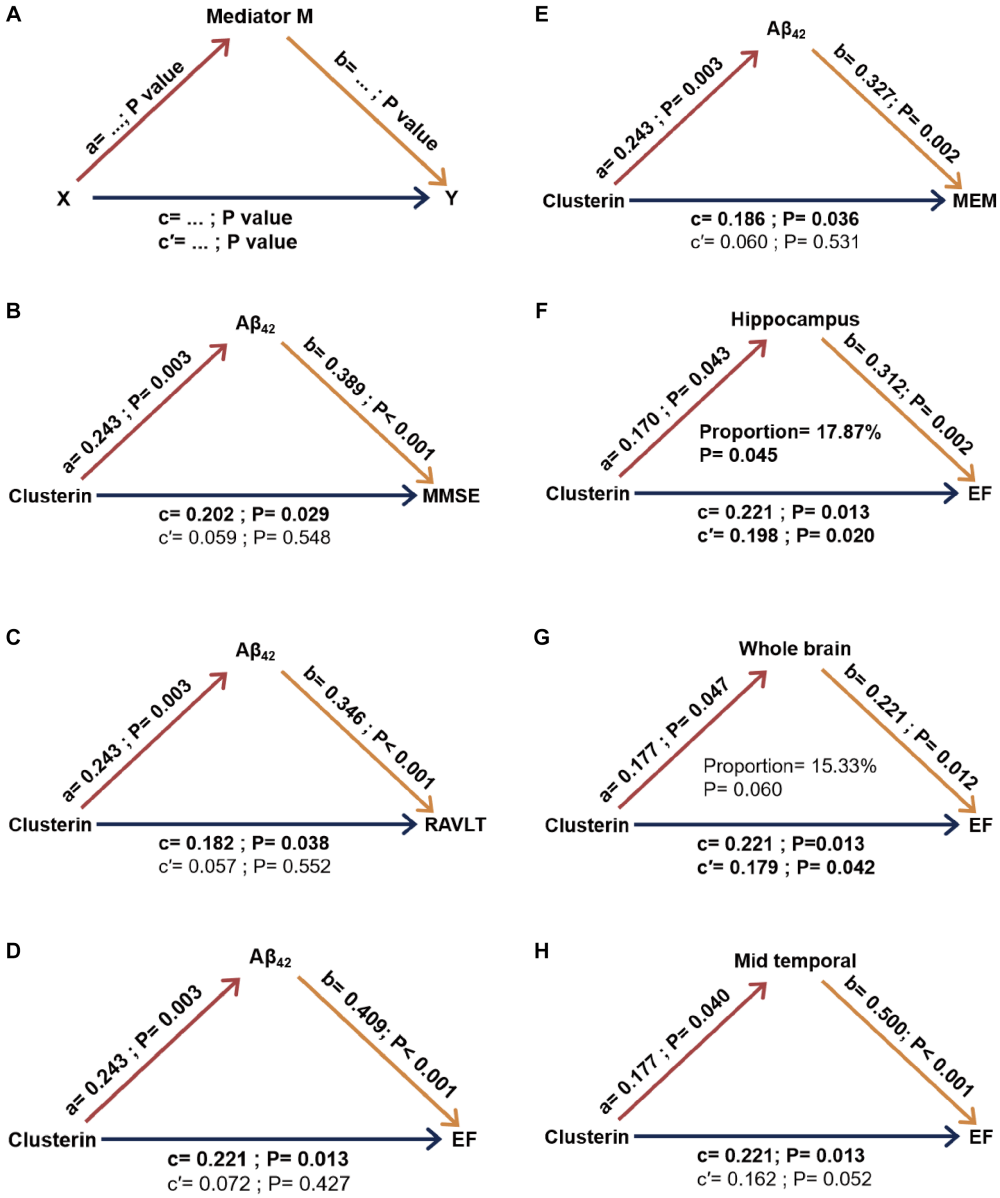
Mediation analysis results concerning clusterin’s impact on longitudinal cognition with biomarkers serving as mediators in MCI. The concept of mediation is visually interpreted in part **(A)**. Specific results in part **(B-H)**. a, b, c, c’ were took into account factors such as age, gender, education level, *APOE ε4* carrier status, and intracranial volume. Aβ_42_, Amyloid-β_42_; MMSE, Mini-Mental State Examination; RAVLT, Rey auditory verbal learning test immediate recall; MEM, memory function composite score; EF, executive function composite score. X, independent variable; Y, dependent variable; M, mediating variable; proportion, percentage of mediation (obtained using a bootstrap method). a: clusterin’s effect on biomarker levels. b: biomarkers’ effect on cognitive scores. c: Total effect of clusterin on cognitive scores without mediation. c’: direct effect of clusterin on cognitive scores considering mediation.

Our initial regression studies across various groups unveiled the relationships between the index of pathology and cognitive parameters within a model that accounted for factors such as age, gender, education level, and *APOE ε4* status. We delved into whether the connection between baseline CSF clusterin and cognitive parameters was influenced by factors such as CSF Aβ_42_, tau-related pathology, and neuroimaging variables. For a graphical representation of the mediation concept, see Figure 4A. There is no evidence found to suggest that the effects of CSF clusterin on cognitive impairments are mediated by its modulation of other AD markers in cross-sectional.

Figure 4 demonstrates the ultimate models of MCI participants, wherein some mediators exhibited a minimum of the trend toward significance in the mediation pathway. Our findings suggest that the association between baseline clusterin and cognitive impairment, specifically executive function (EF), was partially mediated by longitudinal neurodegeneration as indicated by hippocampus volume, accounting for approximately 17.84% of the total effect. As for other possible mediation relationships, no meaningful mediating effects were observed in models with these biomarkers as mediating variables (CSF Aβ, tau pathology, whole brain volume, and mid-temporal volume) (Figure 4).

An estimation of hazard ratios (HRs) was performed using Cox proportional hazards regression analysis based on tertiles of baseline clustering. However, no significant results was observed in the analysis (Supplementary Table S7). We examined the correlation between CSF clusterin and biomarkers in both A− and A+ subjects. Our analysis showed a significant association between CSF clusterin and both CSF Aβ and tau pathology in the A+ subgroup, which is similar to the results observed in the whole MCI population (Supplementary Tables S11–S13).

## 4. Discussion

Current cohort studies of CSF clusterin were few and most have focused on plasma clusterin (Lidström et al., 1998; Thambisetty et al., 2010). The association between clusterin and AD pathology is unclear, and we used this study, a large longitudinal cohort of non-demented individuals was used to comprehensively investigate the predictive value of CSF clusterin in relation to various indicators of AD pathology. The findings revealed that baseline CSF clusterin was a predictor of longitudinal cognitive impairment in individuals with MCI, and that baseline CSF clusterin concentrations were more predictive in females under the age of 65. These results provide valuable insights into the potential role of CSF clusterin in the pathogenesis and progression of AD.

A significant negative correlation was found between plasma clusterin concentration and cognitive score in a study investigating the association between plasma clusterin and AD cognition (Thambisetty et al., 2010). However, in contrast to their result, our findings revealed a significant association between CSF clusterin levels and executive function in the MCI population in cross-sectional. In a study of plasma clusterin levels, lower clusterin groups were also found to have lower cognitive scores, which was consistent with our findings (Romagnoli et al., 2021).

Our findings suggest that CSF clusterin levels are associated with changes in pathology related to AD: decreased CSF clusterin levels in early AD (MCI) is associated with abnormal Aβ pathology, previous research has suggested that the correlation between clusterin and CSF Aβ_42_ may be related to the specific interaction of clusterin with amyloid. Several mechanisms may underlie these associations. Firstly, clusterin has been found to interact with prefibrillar species, inhibiting amyloid plaque formation in a substrate-dependent manner when the ratio of clusterin to Aβ peptide is relatively high (Yerbury et al., 2007). This association is more likely to be detected in the early stages of AD, consistent with our findings. Secondly, clusterin is sensitive to oxidative stress and has been reported to have several oxidative stress-related loci on its gene promoter. It is involved in various cellular stress responses such as the acute myocardial injury response and enhanced antioxidation in tumor cells. In the context of AD pathology, Aβ can trigger the generation of reactive oxygen species (ROS) by activating NMDA receptors. This stressful environment may lead to the upregulation of clusterin (Tiwari et al., 2019; Kalvaityte et al., 2022).

Rik Ossenkoppele et al. suggested that tau protein aggregation plays a pivotal role in the development of clinical AD. Their study revealed a strong link between a high burden of tau protein and cognitive decline. *In vitro* studies further support this relationship, showing that tau pathology can induce synaptic loss and reduce neural network function, thereby emphasizing the close association between tau protein pathology, neurodegeneration, and the onset of clinical symptoms (Ossenkoppele et al., 2022). Hyperphosphorylated tau can impair synaptic function and increase excitotoxicity and is also implicated in Aβ-induced neuronal death (Leschik et al., 2007; Khan and Bloom, 2016).

The causal relationship between increased CSF clusterin aggregates and tau protein pathology, changes in neurons is unclear. Patricia Yuste-Checa et al. found elevated CSF clusterin levels in individuals with tau pathology and Manifestations of neurodegeneration (Martin-Rehrmann et al., 2005; Yuste-Checa et al., 2021).

In our cross-sectional study, the tau pathology and neurodegeneration are associated with increased CSF clusterin levels. These findings are consistent with previous studies (Martin-Rehrmann et al., 2005; Deming et al., 2016). The cross-sectional correlation between tau and clusterin may suggest an upregulated response of clusterin to tau pathology (Nuutinen et al., 2009; Wojtas et al., 2020). Furthermore, this relationship is observed in cognitively normal populations and clusterin correlates with age, suggesting that this effect may be associated with normal aging (O'Bryan et al., 1993).

AD pathology typically begins in the temporal lobe region, particularly the medial temporal lobe (MTL), this particular area is involved in learning memory, stress and emotional response, consisting of structures such as the parahippocampal cortices, hippocampus and entorhinal (Jin et al., 2022), then spreads to the limbic regions of the medial and inferior temporal lobes, the posterior cingulate cortex, and finally to the isocortical areas of the brain (Serrano-Pozo et al., 2011). Clusterin was associated with several brain regions associated with cognition in our study, such as the hippocampus and middle temporal gyrus, which are mentioned in the Results section.

A previous meta-analysis showed that the mean volume of the hippocampus was smaller in the MCI population compared to the normal elderly group, which is consistent with our finding (Shi et al., 2009). Hippocampal atrophy has been found in people with MCI and has been linked to cognitive decline in a meta-analysis, which is consistent with our findings (Nickl-Jockschat et al., 2012).

Higher levels of clusterin at baseline correlate with a baseline volume of the hippocampus, similar associated brain regions also encompass the middle temporal gyrus, which primarily functions in explicit memory, language processing, and social cognition (Xu et al., 2019) suggesting that processing clusterin may be involved in the protection of neurons in relevant brain regions. Previous research indicates that clusterin may have neuroprotective properties, and in AD, reduced levels of this protein may be linked to neuronal degeneration and cell death (Giannakopoulos et al., 1998).

We identified a significant positive correlation between executive function (EF) and the level of clusterin. This finding suggests that higher levels of clusterin are associated with better executive functioning. The positive correlation with EF might be grounded on the neuroprotective role of clusterin, where higher levels could be indicative of a better neuronal health and, consequently, better cognitive performance, particularly in tasks requiring executive control.

Only a few cohort studies have systematically investigated longitudinal changes in biomarkers within MCI populations. A previous study identified a correlation between clusterin levels and rates of change in cognitively relevant brain regions (Thambisetty et al., 2012). Their research demonstrated that elevated clusterin levels correlated with a slower pace of brain atrophy. We observed similar results to theirs, particularly in the region of the temporal lobe and hippocampus. Our results echo previous research findings, showcasing that elevated levels of clusterin are associated with a slower rate of brain atrophy, notably in critical regions implicated in cognitive functions such as the hippocampus and the broader brain structure. The results indicate that higher clusterin levels are associated with a slower decline in the volume of both the hippocampus and the whole brain over time, suggesting a potential protective role of clusterin in preserving brain volume. Additionally, we found that clusterin levels were associated with cognitive changes in the MCI population, especially in memory and executive function. Over time, higher levels of clusterin proteins are associated with a slower decline in cognitive scores. Interestingly, we found a correlation between clusterin and longitudinal changes in tau pathology. Higher levels of clusterin were associated with a slower accumulation of tau pathology over time. The interaction mechanism between clusterin and tau pathology remains a subject of debate. One possible explanation is that the body responds to stressors, such as phosphorylated tau protein aggregation or cerebrovascular disease induction, by increasing clusterin concentration. The presence of clusterin impeded the formation and accumulation of tau aggregates, as reported in a cellular experiment (Mok et al., 2018; Dhiman et al., 2019; Wojtas et al., 2020; Yuste-Checa et al., 2022).

Based on these results, we conducted mediation analyses to determine which factors mediate the relationship between clusterin levels and cognitive performance. In the CN population, we did not observe a significant effect. However, in the mild cognitive impairment (MCI) population, our analysis suggested that the impact of clusterin on cognitive measures was partially mediated by neurodegeneration, specifically in the hippocampus. Previous research has also demonstrated that hippocampal atrophy can be observed in individuals with MCI and is associated with cognitive decline. Moreover, hippocampal atrophy has been established as a sensitive and specific marker for early detection of AD (Hampel et al., 2005). While we conducted a mediation analysis with AD biomarkers as intermediaries, and the results indicate that clusterin does not exert its influence on brain regions through the major biomarkers, including tau protein (Supplementary Table S10).

Our study’s findings imply that clusterin may exhibit neuroprotective effects during the initial stages of AD. Nonetheless, additional research is required to validate these findings and elucidate the underlying mechanisms connecting clusterin, hippocampal atrophy, and cognitive function.

Several limitations to our study should be acknowledged. Firstly, the lack of complete follow-up in our cohort may limit the reliability of our findings. Secondly, our analyses were based on CSF protein measurements which may not be as accurate as PET imaging data. Thirdly, there is variability in the results of different studies on clusterin which may require clarification through larger and more diverse cohorts. Lastly, the sampling of CSF clusterin is an invasive procedure that is not accessible to all patients, which may limit the generalizability of our findings. Due to the absence of long-term follow-up data on clusterin protein, we are unable to provide a comprehensive explanation for causality.

To sum up, our study has revealed a link between clusterin and important biomarkers of AD and has evaluated the protein’s long-term effect on the disease’s pathology using a longitudinal cohort. Our findings pave the way for further investigations into the complex role of this glycoprotein.

## Data availability statement

The original contributions presented in the study are included in the article/Supplementary material, further inquiries can be directed to the corresponding authors.

## Ethics statement

The studies involving human participants were reviewed and approved by the Alzheimer’s Disease Neuroimaging Initiative (ADNI). All participants were made aware of the study’s aim and granted their approval via signed consent forms.

## Author contributions

HW: Data curation, Formal analysis, Investigation, Methodology, Software, Writing – original draft. L-ZM: Formal analysis, Conceptualization, Data curation, Methodology, Writing – review & editing. Z-HS: Data curation, Formal analysis, Methodology, Supervision, Validation, Writing – original draft. J-YL: Methodology, Writing – original draft, Data curation. W-YY: Data curation, Writing – original draft. FG: Data curation, Writing – original draft. WZ: Conceptualization, Supervision, Writing – review & editing. LT: Conceptualization, Supervision, Writing – review & editing, Project administration.

## References

[ref1] Aisen P. S. Petersen R. C. Donohue M. C. Gamst A. Raman R. Thomas R. G.. (2010). Clinical core of the Alzheimer's disease neuroimaging initiative: Progress and plans. Alzheimers Dement. 6, 239–246. doi: 10.1016/j.jalz.2010.03.006, PMID: 20451872 PMC2867843

[ref2] Chen Y. Qian X. Zhang Y. Su W. Huang Y. Wang X.. (2022). Prediction models for conversion from mild cognitive impairment to Alzheimer's disease: a systematic review and meta-analysis. Front. Aging Neurosci. 14:840386. doi: 10.3389/fnagi.2022.840386, PMID: 35493941 PMC9049273

[ref3] Crane P. K. Carle A. Gibbons L. E. Insel P. Mackin R. S. Gross A.. (2012). Development and assessment of a composite score for memory in the Alzheimer's disease neuroimaging initiative (ADNI). Brain Imaging Behav. 6, 502–516. doi: 10.1007/s11682-012-9186-z, PMID: 22782295 PMC3806057

[ref4] Deming Y. Xia J. Cai Y. Lord J. Holmans P. Bertelsen S.. (2016). A potential endophenotype for Alzheimer's disease: cerebrospinal fluid clusterin. Neurobiol. Aging 37, 208.e1–208.e9. doi: 10.1016/j.neurobiolaging.2015.09.009 PMC5118651 26545630

[ref5] Dhiman K. Blennow K. Zetterberg H. Martins R. N. Gupta V. B. (2019). Cerebrospinal fluid biomarkers for understanding multiple aspects of Alzheimer’s disease pathogenesis. Cell. Mol. Life Sci. 76, 1833–1863. doi: 10.1007/s00018-019-03040-5, PMID: 30770953 PMC11105672

[ref6] Djeu J. Y. Wei S. (2009). Clusterin and chemoresistance. Adv. Cancer Res. 105, 77–92. doi: 10.1016/S0065-230X(09)05005-2, PMID: 19879424 PMC3889866

[ref7] Duong S. Patel T. Chang F. (2017). Dementia: what pharmacists need to know. Can. Pharm. J. (Ott) 150, 118–129. doi: 10.1177/1715163517690745, PMID: 28405256 PMC5384525

[ref8] Falahati F. Ferreira D. Muehlboeck J. S. Eriksdotter M. Simmons A. Wahlund L. O.. (2017). Monitoring disease progression in mild cognitive impairment: associations between atrophy patterns, cognition, APOE and amyloid. Neuroimage Clin. 16, 418–428. doi: 10.1016/j.nicl.2017.08.014, PMID: 28879083 PMC5573795

[ref9] Folstein M. F. Folstein S. E. McHugh P. R. (1975). “Mini-mental state”: a practical method for grading the cognitive state of patients for the clinician. J. Psychiatr. Res. 12, 189–198. doi: 10.1016/0022-3956(75)90026-6 1202204

[ref10] Foster E. M. Dangla-Valls A. Lovestone S. Ribe E. M. Buckley N. J. (2019). Clusterin in Alzheimer's disease: mechanisms, genetics, and lessons from other pathologies. Front. Neurosci. 13:164. doi: 10.3389/fnins.2019.00164, PMID: 30872998 PMC6403191

[ref11] Giannakopoulos P. Kövari E. French L. E. Viard I. Hof P. R. Bouras C. (1998). Possible neuroprotective role of clusterin in Alzheimer’s disease: a quantitative immunocytochemical study. Acta Neuropathol. 95, 387–394. doi: 10.1007/s004010050815, PMID: 9560017

[ref12] Giau V. V. Bagyinszky E. An S. S. A. (2019). Potential fluid biomarkers for the diagnosis of mild cognitive impairment. Int. J. Mol. Sci. 20:4149. doi: 10.3390/ijms20174149, PMID: 31450692 PMC6747411

[ref13] Gibbons L. E. Carle A. C. Mackin R. S. Harvey D. Mukherjee S. Insel P.. (2012). A composite score for executive functioning, validated in Alzheimer's disease neuroimaging initiative (ADNI) participants with baseline mild cognitive impairment. Brain Imaging Behav. 6, 517–527. doi: 10.1007/s11682-012-9176-1, PMID: 22644789 PMC3684181

[ref14] Hampel H. Bürger K. Pruessner J. C. Zinkowski R. DeBernardis J. Kerkman D.. (2005). Correlation of cerebrospinal fluid levels of tau protein phosphorylated at threonine 231 with rates of hippocampal atrophy in Alzheimer disease. Arch. Neurol. 62, 770–773. doi: 10.1001/archneur.62.5.770, PMID: 15883264

[ref15] Harold D. Abraham R. Hollingworth P. Sims R. Gerrish A. Hamshere M. L.. (2009). Genome-wide association study identifies variants at CLU and PICALM associated with Alzheimer's disease. Nat. Genet. 41, 1088–1093. doi: 10.1038/ng.440, PMID: 19734902 PMC2845877

[ref16] Jin W. Jie F. Wenwei Z. Bin Z. Chen S. Wei S.. (2022). The medial temporal lobe structure and function support positive affect. Neuropsychologia 176:108373. doi: 10.1016/j.neuropsychologia.2022.108373, PMID: 36167193

[ref17] Kalvaityte U. Matta C. Bernotiene E. Pushparaj P. N. Kiapour A. M. Mobasheri A. (2022). Exploring the translational potential of clusterin as a biomarker of early osteoarthritis. J. Orthop. Translat. 32, 77–84. doi: 10.1016/j.jot.2021.10.001, PMID: 34976733 PMC8671091

[ref18] Karran E. De Strooper B. (2022). The amyloid hypothesis in Alzheimer disease: new insights from new therapeutics. Nat. Rev. Drug Discov. 21, 306–318. doi: 10.1038/s41573-022-00391-w, PMID: 35177833

[ref19] Karran E. Mercken M. De Strooper B. (2011). The amyloid cascade hypothesis for Alzheimer's disease: an appraisal for the development of therapeutics. Nat. Rev. Drug Discov. 10, 698–712. doi: 10.1038/nrd3505, PMID: 21852788

[ref20] Kennedy J. J. Abbatiello S. E. Kim K. Yan P. Whiteaker J. R. Lin C.. (2014). Demonstrating the feasibility of large-scale development of standardized assays to quantify human proteins. Nat. Methods 11, 149–155. doi: 10.1038/nmeth.2763, PMID: 24317253 PMC3922286

[ref21] Khan S. S. Bloom G. S. (2016). Tau: the Center of a Signaling Nexus in Alzheimer's disease. Front. Neurosci. 10:31. doi: 10.3389/fnins.2016.00031, PMID: 26903798 PMC4746348

[ref22] Leschik J. Welzel A. Weissmann C. Eckert A. Brandt R. (2007). Inverse and distinct modulation of tau-dependent neurodegeneration by presenilin 1 and amyloid-beta in cultured cortical neurons: evidence that tau phosphorylation is the limiting factor in amyloid-beta-induced cell death. J. Neurochem. 101, 1303–1315. doi: 10.1111/j.1471-4159.2006.04435.x, PMID: 17298384

[ref23] Lidström A. M. Bogdanovic N. Hesse C. Volkman I. Davidsson P. Blennow K. (1998). Clusterin (apolipoprotein J) protein levels are increased in hippocampus and in frontal cortex in Alzheimer's disease. Exp. Neurol. 154, 511–521. doi: 10.1006/exnr.1998.6892, PMID: 9878186

[ref24] Liu Y. Zhang H. Zhong X. Li Z. Zetterberg H. Li L. (2022). Isotopic N,N-dimethyl leucine tags for absolute quantification of clusterin and apolipoprotein E in Alzheimer's disease. J. Proteome 257:104507. doi: 10.1016/j.jprot.2022.104507, PMID: 35124278 PMC8916911

[ref25] Ma L.-Z. Zhang C. Wang H. Ma Y.-H. Shen X.-N. Wang J.. (2021). Serum neurofilament dynamics predicts cognitive progression in de novo Parkinson’s disease. J. Parkinsons Dis. 11, 1117–1127. doi: 10.3233/JPD-212535, PMID: 33935105

[ref26] Mangialasche F. Westman E. Kivipelto M. Muehlboeck J. S. Cecchetti R. Baglioni M.. (2013). Classification and prediction of clinical diagnosis of Alzheimer's disease based on MRI and plasma measures of α−/γ-tocotrienols and γ-tocopherol. J. Intern. Med. 273, 602–621. doi: 10.1111/joim.12037, PMID: 23343471

[ref27] Martin-Rehrmann M. D. Hoe H.-S. Capuani E. M. Rebeck G. W. (2005). Association of apolipoprotein J-positive β-amyloid plaques with dystrophic neurites in alzheimer’s disease brain. Neurotox. Res. 7, 231–241. doi: 10.1007/BF03036452, PMID: 15897157

[ref28] Mok S.-A. Condello C. Freilich R. Gillies A. Arhar T. Oroz J.. (2018). Mapping interactions with the chaperone network reveals factors that protect against tau aggregation. Nat. Struct. Mol. Biol. 25, 384–393. doi: 10.1038/s41594-018-0057-1, PMID: 29728653 PMC5942583

[ref29] Nickl-Jockschat T. Kleiman A. Schulz J. B. Schneider F. Laird A. R. Fox P. T.. (2012). Neuroanatomic changes and their association with cognitive decline in mild cognitive impairment: a meta-analysis. Brain Struct. Funct. 217, 115–125. doi: 10.1007/s00429-011-0333-x, PMID: 21667303 PMC4791066

[ref30] Nuutinen T. Suuronen T. Kauppinen A. Salminen A. (2009). Clusterin: a forgotten player in Alzheimer's disease. Brain Res. Rev. 61, 89–104. doi: 10.1016/j.brainresrev.2009.05.007, PMID: 19651157

[ref31] O'Bryan M. K. Cheema S. S. Bartlett P. F. Murphy B. F. Pearse M. J. (1993). Clusterin levels increase during neuronal development. J. Neurobiol. 24, 421–432. doi: 10.1002/neu.480240402, PMID: 8515248

[ref32] Ossenkoppele R. van der Kant R. Hansson O. (2022). Tau biomarkers in Alzheimer's disease: towards implementation in clinical practice and trials. Lancet Neurol. 21, 726–734. doi: 10.1016/S1474-4422(22)00168-5, PMID: 35643092

[ref33] Pooler A. M. Polydoro M. Maury E. A. Nicholls S. B. Reddy S. M. Wegmann S.. (2015). Amyloid accelerates tau propagation and toxicity in a model of early Alzheimer's disease. Acta Neuropathol. Commun. 3:14. doi: 10.1186/s40478-015-0199-x, PMID: 25853174 PMC4371800

[ref34] Romagnoli T. Ortolani B. Sanz J. M. Trentini A. Seripa D. Nora E. D.. (2021). Serum apo J as a potential marker of conversion from mild cognitive impairment to dementia. J. Neurol. Sci. 427:117537. doi: 10.1016/j.jns.2021.117537, PMID: 34147956

[ref35] Rosselli M. Uribe I. V. Ahne E. Shihadeh L. (2022). Culture, ethnicity, and level of education in Alzheimer’s disease. Neurotherapeutics 19, 26–54. doi: 10.1007/s13311-022-01193-z, PMID: 35347644 PMC8960082

[ref36] Schindler S. E. Gray J. D. Gordon B. A. Xiong C. Batrla-Utermann R. Quan M.. (2018). Cerebrospinal fluid biomarkers measured by Elecsys assays compared to amyloid imaging. Alzheimers Dement. 14, 1460–1469. doi: 10.1016/j.jalz.2018.01.013, PMID: 29501462 PMC6119652

[ref37] Serrano-Pozo A. Frosch M. P. Masliah E. Hyman B. T. (2011). Neuropathological alterations in Alzheimer disease. Cold Spring Harb. Perspect. Med. 1:a006189. doi: 10.1101/cshperspect.a006189, PMID: 22229116 PMC3234452

[ref38] Shaw L. M. Vanderstichele H. Knapik-Czajka M. Clark C. M. Aisen P. S. Petersen R. C.. (2009). Cerebrospinal fluid biomarker signature in Alzheimer's disease neuroimaging initiative subjects. Ann. Neurol. 65, 403–413. doi: 10.1002/ana.21610, PMID: 19296504 PMC2696350

[ref39] Shi F. Liu B. Zhou Y. Yu C. Jiang T. (2009). Hippocampal volume and asymmetry in mild cognitive impairment and Alzheimer's disease: meta-analyses of MRI studies. Hippocampus 19, 1055–1064. doi: 10.1002/hipo.20573, PMID: 19309039

[ref40] Talwar P. Kushwaha S. Chaturvedi M. Mahajan V. (2021). Systematic review of different neuroimaging correlates in mild cognitive impairment and Alzheimer's disease. Clin. Neuroradiol. 31, 953–967. doi: 10.1007/s00062-021-01057-7, PMID: 34297137

[ref41] Tang L. Wang Z. B. Ma L. Z. Cao X. P. Tan L. Tan M. S. (2022). Dynamic changes of CSF clusterin levels across the Alzheimer's disease continuum. BMC Neurol. 22:508. doi: 10.1186/s12883-022-03038-w, PMID: 36581903 PMC9801612

[ref42] Thambisetty M. An Y. Kinsey A. Koka D. Saleem M. Guntert A.. (2012). Plasma clusterin concentration is associated with longitudinal brain atrophy in mild cognitive impairment. NeuroImage 59, 212–217. doi: 10.1016/j.neuroimage.2011.07.056, PMID: 21824521 PMC3425349

[ref43] Thambisetty M. Simmons A. Velayudhan L. Hye A. Campbell J. Zhang Y.. (2010). Association of Plasma Clusterin Concentration with Severity, pathology, and progression in Alzheimer disease. Arch. Gen. Psychiatry 67, 739–748. doi: 10.1001/archgenpsychiatry.2010.78, PMID: 20603455 PMC3111021

[ref44] Tiwari S. Atluri V. Kaushik A. Yndart A. Nair M. (2019). Alzheimer's disease: pathogenesis, diagnostics, and therapeutics. Int. J. Nanomedicine 14, 5541–5554. doi: 10.2147/IJN.S200490, PMID: 31410002 PMC6650620

[ref45] Veitch D. P. Weiner M. W. Aisen P. S. Beckett L. A. Cairns N. J. Green R. C.. (2019). Understanding disease progression and improving Alzheimer's disease clinical trials: recent highlights from the Alzheimer's disease neuroimaging initiative. Alzheimers Dement. 15, 106–152. doi: 10.1016/j.jalz.2018.08.005, PMID: 30321505

[ref46] Veitch D. P. Weiner M. W. Aisen P. S. Beckett L. A. DeCarli C. Green R. C.. (2022). Using the Alzheimer's disease neuroimaging initiative to improve early detection, diagnosis, and treatment of Alzheimer's disease. Alzheimers Dement. 18, 824–857. doi: 10.1002/alz.12422, PMID: 34581485 PMC9158456

[ref47] Wang X. Huang W. Su L. Xing Y. Jessen F. Sun Y.. (2020). Neuroimaging advances regarding subjective cognitive decline in preclinical Alzheimer's disease. Mol. Neurodegener. 15:55. doi: 10.1186/s13024-020-00395-3, PMID: 32962744 PMC7507636

[ref48] Wojtas A. M. Carlomagno Y. Sens J. P. Kang S. S. Jensen T. D. Kurti A.. (2020). Clusterin ameliorates tau pathology in vivo by inhibiting fibril formation. Acta Neuropathol. Commun. 8:210. doi: 10.1186/s40478-020-01079-1, PMID: 33261653 PMC7708249

[ref49] Wu Z. C. Yu J. T. Li Y. Tan L. (2012). Clusterin in Alzheimer's disease. Adv. Clin. Chem. 56, 155–173. doi: 10.1016/b978-0-12-394317-0.00011-x 22397031

[ref50] Xu J. Lyu H. Li T. Xu Z. Fu X. Jia F.. (2019). Delineating functional segregations of the human middle temporal gyrus with resting-state functional connectivity and coactivation patterns. Hum. Brain Mapp. 40, 5159–5171. doi: 10.1002/hbm.24763, PMID: 31423713 PMC6865466

[ref51] Xu J. Yuan M. Q. Fang Y. (2022). Research on predicting the risk of mild cognitive impairment in the elderly based on the joint model. Zhonghua Liu Xing Bing Xue Za Zhi 43, 269–276. doi: 10.3760/cma.j.cn112338-20210620-00484, PMID: 35184495

[ref52] Yerbury J. J. Poon S. Meehan S. Thompson B. Kumita J. R. Dobson C. M.. (2007). The extracellular chaperone clusterin influences amyloid formation and toxicity by interacting with prefibrillar structures. FASEB J. 21, 2312–2322. doi: 10.1096/fj.06-7986com, PMID: 17412999

[ref53] Yu Q. Mai Y. Ruan Y. Luo Y. Zhao L. Fang W.. (2021). An MRI-based strategy for differentiation of frontotemporal dementia and Alzheimer's disease. Alzheimers Res. Ther. 13:23. doi: 10.1186/s13195-020-00757-5, PMID: 33436059 PMC7805212

[ref54] Yuste-Checa P. Bracher A. Hartl F. U. (2022). The chaperone Clusterin in neurodegeneration-friend or foe? BioEssays 44:e2100287. doi: 10.1002/bies.202100287 35521968

[ref55] Yuste-Checa P. Trinkaus V. A. Riera-Tur I. Imamoglu R. Schaller T. F. Wang H.. (2021). The extracellular chaperone Clusterin enhances tau aggregate seeding in a cellular model. Nat. Commun. 12:4863. doi: 10.1038/s41467-021-25060-1, PMID: 34381050 PMC8357826

