# Supplementary material for: Association between cerebrospinal fluid clusterin and biomarkers of Alzheimer’s disease pathology in mild cognitive impairment: a longitudinal cohort study
Source: Front Aging Neurosci. 2023 Oct 24;15:1256389. doi: 10.3389/fnagi.2023.1256389 (PMC10629112; doi:10.3389/fnagi.2023.1256389)
Supplement: Supplementary file 2 [file Data_Sheet_2.docx]

Supplementary Material

Supplementary Figures and Tables

**Supplementary Figure 1.** The Quantile-Quantile plot of clusterin


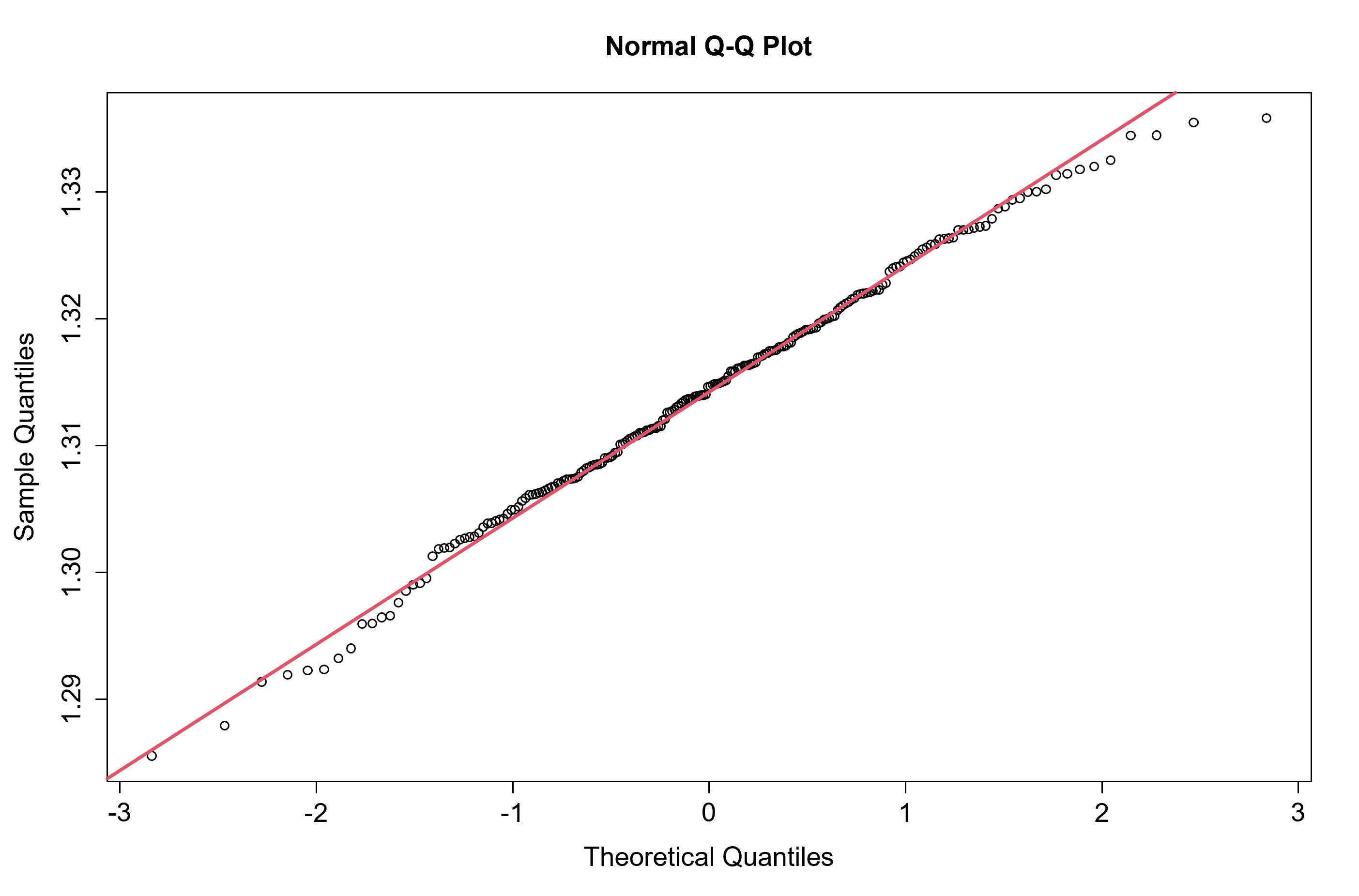


Sample quantiles were plotted follow those theoretical quantiles under the null hypothesis (x-axis). The Q-Q plot shows that log10-transformed CSF clusterin data conforms to the Normal Distribution.

**Abbreviations**: CSF, cerebrospinal fluid;

**Supplementary Figure 2.** log10-transformed CSF clusterin was positively correlated with age in


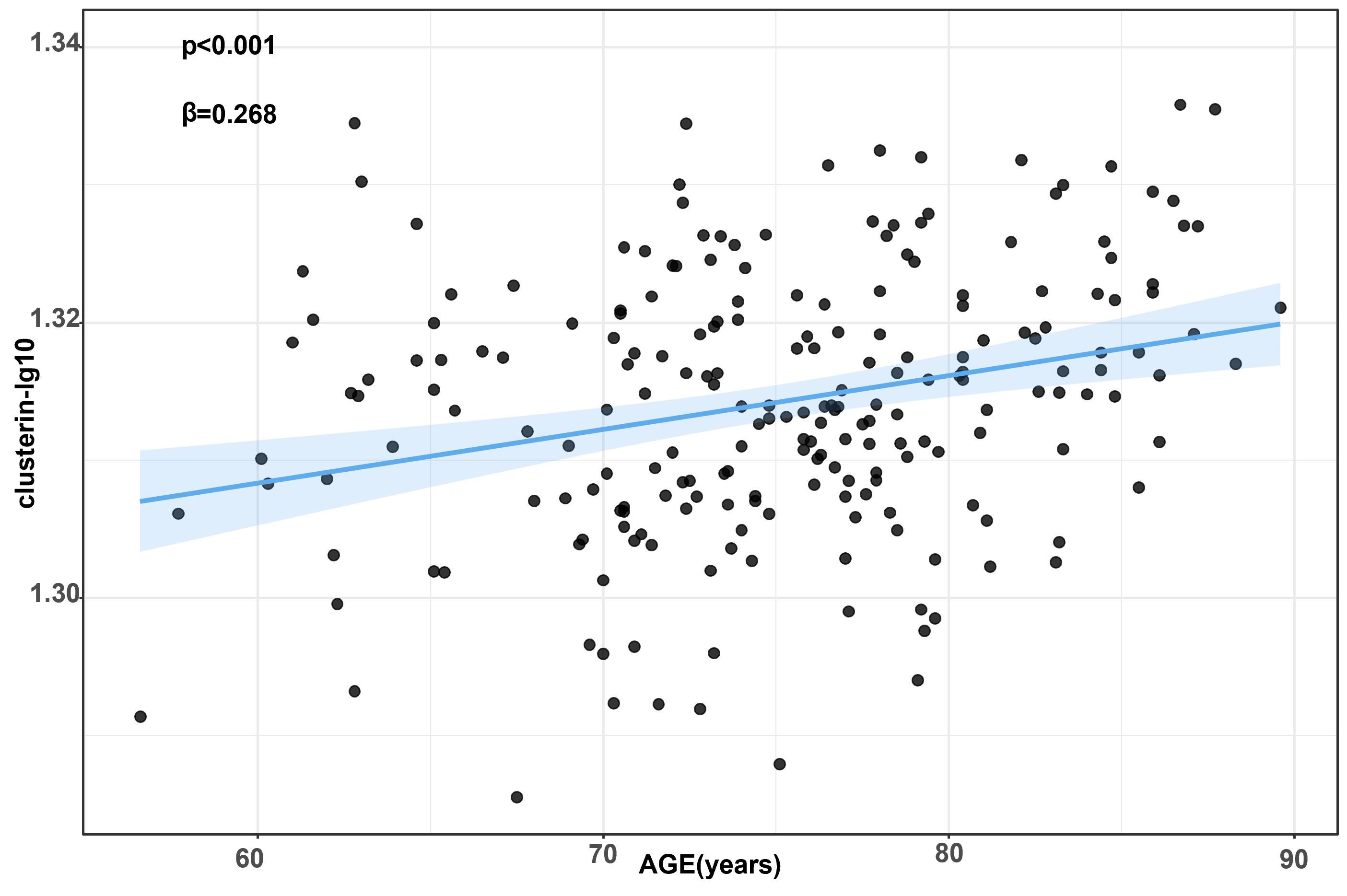


Associations of log10-transformed CSF clusterin and age. The normalized regression coefficients (β) and p values were computed by linear regression.

**Supplementary Figure 3.** log10-transformed CSF clusterin was positively correlated with age


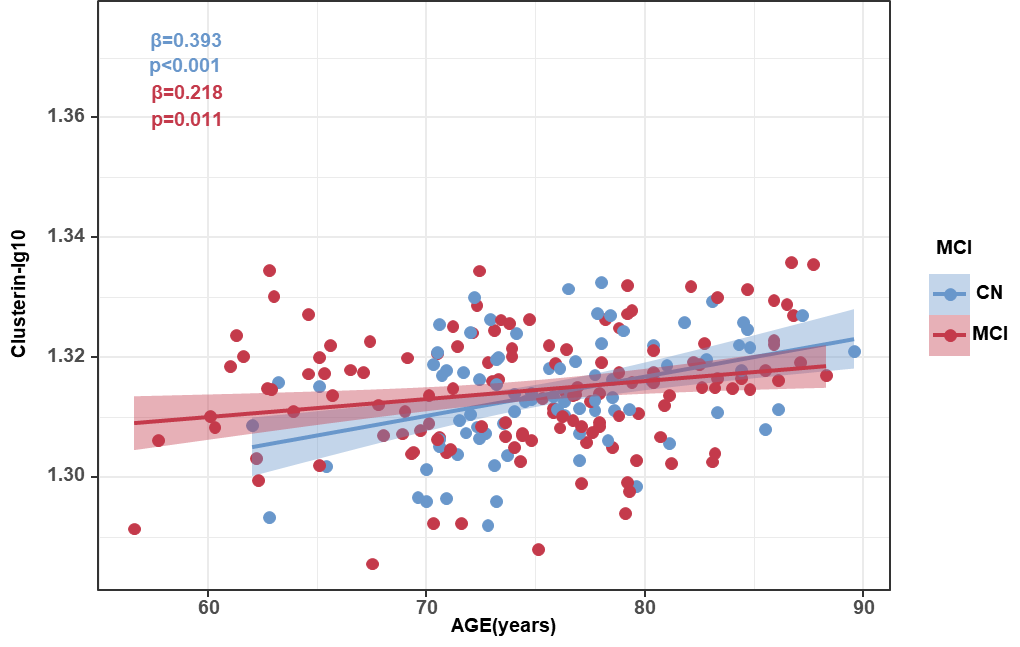


Associations of log10-transformed CSF clusterin and age. The normalized regression coefficients (β) and p values were computed by linear regression.

**Supplementary Table 1.** Follow-up data of longitudinal CSF, neuroimaging, and cognitive symptoms

| **study visit** | **bl** | **m24** | **m48** | **m72** | **m96** | **m120** | **m144** | **m168** | **m180** |
| --- | --- | --- | --- | --- | --- | --- | --- | --- | --- |
| Aβ_42_ | 188 | 53 | 44 | 16 | 4 | 3 | - | - | - |
| T-tau | 216 | 62 | 51 | 18 | 7 | 4 | - | - | - |
| P-tau | 216 | 62 | 51 | 18 | 7 | 4 | - | - | - |
| ADAS11 | 205 | 185 | 96 | 83 | 52 | 29 | 18 | 4 | 3 |
| ADAS13 | 211 | 191 | 98 | 87 | 51 | 30 | 19 | 4 | 3 |
| ADASQ4 | 220 | 199 | 106 | 92 | 57 | 31 | 19 | 4 | 3 |
| MMSE | 206 | 186 | 98 | 83 | 51 | 29 | 19 | 4 | 3 |
| RAVLT immediate | 220 | 197 | 107 | 91 | 55 | 31 | 19 | 4 | 3 |
| MEM | 220 | 199 | 108 | 92 | 58 | 31 | 19 | 4 | 3 |
| EF | 220 | 199 | 108 | 92 | 58 | 31 | 19 | 4 | 3 |
| Ventricles | 216 | 176 | 88 | 91 | 14 | 4 | - | - | - |
| Hippocampus | 181 | 144 | 74 | 53 | 16 | 5 | - | - | - |
| Whole brain | 218 | 179 | 91 | 69 | 15 | 4 | - | - | - |
| Entorhinal | 181 | 144 | 73 | 56 | 12 | 3 | - | - | - |
| Fusiform | 181 | 144 | 73 | 56 | 12 | 3 | - | - | - |
| Mid temporal | 181 | 144 | 73 | 56 | 12 | 3 | - | - | - |

The data are number of data points at each study visit for different measures. **Abbreviations**: Aβ42, Amyloid-β42; T -tau, Total tau; P-tau, Phosphorylated tau; ADAS, Alzheimer's disease assessment scale-cognitive; ADASQ4 , ADAS delayed word recall; MMSE, Mini-Mental State Examination; RAVLT immediate, Rey Auditory Verbal Learning Test immediate recall; MEM, memory function composite score; EF, executive function composite score.

**Supplementary Table 2.** Association of baseline clusterin with CSF biomarkers, neuroimaging and cognitive data observed among participants.

| **Baseline Measure** | β | P |
| --- | --- | --- |
| Aβ_42_ | **0.212** | **0.001** |
| T-tau | **0.423** | **<0.001** |
| P-tau | **0.366** | **<0.001** |
| ADAS11 | -0.058 | 0.411 |
| ADAS13 | -0.017 | 0.806 |
| ADASQ4 | 0.015 | 0.825 |
| MMSE | 0.110 | 0.126 |
| RAVLT immediate | -0.009 | 0.898 |
| MEM | 0.012 | 0.861 |
| EF | 0.085 | 0.227 |
| Ventricles | **-0.169** | **0.005** |
| Hippocampus | 0.004 | 0.416 |
| Whole brain | 0.415 | 0.803 |
| Entorhinal | 0.079 | 0.272 |
| Fusiform | 0.105 | 0.118 |
| Mid temporal | **0.146** | **0.033** |

Association of baseline clusterin with CSF biomarkers, neuroimaging and cognitive data observed among participants. All analyses were corrected for age, gender, educational level, *APOEε4* status, and intracranial volume*.* **Abbreviations**: Aβ42, Amyloid-β42; T-tau, Total tau; P-tau, Phosphorylated tau; ADAS, Alzheimer's disease assessment scale-cognitive; ADASQ4 , ADAS delayed word recall; MMSE, Mini-Mental State Examination; RAVLT immediate, Rey Auditory Verbal Learning Test immediate recall; MEM, memory function composite score; EF, executive function composite score.

**Supplementary Table 3.** Linear mixed-effects models for longitudinal CSF biomarkers, neuroimaging, and cognitive symptom severity in participants.

| **Baseline Measure** | β | P |
| --- | --- | --- |
| Aβ_42_ | -0.013 | 0.135 |
| T-tau | **-0.021** | **0.006** |
| P-tau | **-0.019** | **0.008** |
| ADAS11 | -0.005 | 0.618 |
| ADAS13 | -0.004 | 0.656 |
| ADASQ4 | -0.005 | 0.548 |
| MMSE | 0.034 | 0.102 |
| RAVLT immediate | **0.031** | **0.034** |
| MEM | **0.034** | **0.044** |
| EF | **0.033** | **0.042** |
| Ventricles | -0.009 | 0.059 |
| Hippocampus | 0.008 | 0.296 |
| Whole brain | 0.012 | 0.086 |
| Entorhinal | 0.003 | 0.739 |
| Fusiform | 0.012 | 0.255 |
| Mid temporal | **0.024** | **0.033** |

All analyses were corrected for age, gender, educational level, *APOEε4* status, and intracranial volume*.* **Abbreviations**: Aβ42, Amyloid-β42; T-tau, Total tau; P-tau, Phosphorylated tau; ADAS, Alzheimer's disease assessment scale-cognitive; ADASQ4 , ADAS delayed word recall; MMSE, Mini-Mental State Examination; RAVLT immediate, Rey Auditory Verbal Learning Test immediate recall; MEM, memory function composite score; EF, executive function composite score.

| **Supplementary Table 4.** Baseline characteristics based on clusterin CSF levels in participants.. | | | | |
| --- | --- | --- | --- | --- |
| Characteristics | Low(n= 73) | Middle(n= 74) | High(n= 73) | P value |
| AGE (years) | 72.678±6.030 | 75.654±6.382 | 76.916±7.025 | **<0.001** |
| Gender = Male (%) | 37 (50.685) | 44 (59.459) | 54 (73.973) | **0.014** |
| Education (years) | 16.068±2.859 | 15.392±2.779 | 16.164±3.245 | 0.229 |
| *APOE ε4* carriers (%) | 32 (43.8) | 27 (36.5) | 33 (45.2) | 0.514 |
| Aβ42 | 748.015±336.163 | 940.111±416.581 | 873.438±367.926 | **0.011** |
| T-tau | 252.322±100.283 | 274.438±105.548 | 338.782±106.951 | **<0.001** |
| P-tau | 24.548±11.180 | 26.499±12.184 | 32.880±12.893 | **<0.001** |
| ADAS11 | 10.292±5.432 | 9.266±4.778 | 8.991±3.837 | 0.217 |
| ADAS13 | 16.319±8.202 | 14.754±7.246 | 14.552±5.912 | 0.268 |
| ADASQ4 | 5.164±2.872 | 4.919±2.714 | 4.808±2.509 | 0.718 |
| MMSE | 27.644±1.836 | 27.716±1.802 | 27.918±1.824 | 0.642 |
| RAVLT immediate | 35.712±10.641 | 34.446±12.205 | 35.384±8.964 | 0.758 |
| MEM | 0.273±0.821 | 0.282±0.836 | 0.324±0.671 | 0.916 |
| EF | 0.076±0.956 | 0.270±0.790 | 0.153±0.768 | 0.376 |
| Ventricles | 44126.356±25822.924 | 37528.521±17484.322 | 41330.569±20060.609 | 0.177 |
| Hippocampus | 6699.855±1140.622 | 6572.150±1109.375 | 6794.373±1013.761 | 0.537 |
| Whole brain | 998736.740±112226.081 | 991161.425±106361.192 | 1016334.153±97895.276 | 0.341 |
| Entorhinal | 3405.048±753.164 | 3525.233±738.827 | 3612.695±800.322 | 0.326 |
| Fusiform | 16531.790±2635.009 | 16765.800±2131.430 | 17214.881±2180.651 | 0.266 |
| Mid temporal | 18858.790±3390.689 | 18603.033±2526.665 | 19840.831±2586.150 | **0.048** |
| Dementia at follow up(%) | 35 (47.945) | 32 (43.243) | 28 (38.356) | 0.505 |
| Proportion of MCI | 47 (64.4) | 41 (55.4) | 46 (63.0) | 0.485 |
| In bold are highlighted the variables that are significantly different between the group.  Categorical variables are reported as numbers and percentages; continuous variables are reported as means±SDs. Gender, The gender of the participants; Education, Years of education of the participants; CN, cognitively normal; MCI, mild cognitive impairment; APOE, Apolipoprotein E;Aβ42,Amyloid-β42;T-tau,Total tau; P-tau, Phosphorylated tau; ADAS, Alzheimer's disease assessment scale-cognitive;ADASQ4,ADAS delayed word recall; MMSE, Mini Mental State Examination; RAVLT immediate, Rey Auditory Verbal Learning Test immediate recall; MEM, memory function composite score; EF, executive function composite score. | | | | |

| **Supplementary Table 5.**Clinical characteristics of participants based on clusterin CSF levels. | | | |
| --- | --- | --- | --- |
| Characteristics | Low((n= 110) | High(n= 110) | P value |
| AGE (years) | 73.844±5.816 | 76.327±7.300 | **0.006** |
| Gender = Male (%) | 59 (53.636) | 76 (69.091) | **0.027** |
| Education (years) | 15.845±2.864 | 15.900±3.092 | 0.892 |
| *APOE ε4* carriers (%) | 49 (44.545) | 43 (39.091) | 0.494 |
| Aβ_42_ | 780.425±357.292 | 932.083±394.512 | **0.006** |
| T-tau | 262.196±108.035 | 314.573±106.296 | **<0.001** |
| P-tau | 25.553±12.119 | 30.371±12.593 | **0.004** |
| ADAS11 | 9.836±5.158 | 9.194±4.280 | 0.316 |
| ADAS13 | 15.554±7.883 | 14.867±6.454 | 0.481 |
| ADASQ4 | 5.000±2.873 | 4.927±2.515 | 0.842 |
| MMSE | 27.791±1.848 | 27.727±1.791 | 0.796 |
| RAVLT immediate | 35.609±11.191 | 34.745±10.130 | 0.549 |
| MEM | 0.304±0.842 | 0.282±0.709 | 0.835 |
| EF | 0.139±0.912 | 0.194±0.768 | 0.627 |
| Ventricles | 42275.202±23471.044 | 39712.018±19326.895 | 0.380 |
| Hippocampus | 6644.859±1138.165 | 6733.270±1038.061 | 0.586 |
| Whole brain | 994210.174±113199.210 | 1009813.908±97596.988 | 0.277 |
| Entorhinal | 3431.043±731.058 | 3596.854±793.426 | 0.145 |
| Fusiform | 16563.837±2489.853 | 17109.258±2143.384 | 0.117 |
| Mid temporal | 18734.424±3108.198 | 19465.944±2640.005 | 0.090 |
| Dementia at follow up(%) | 51 (46.364) | 44 (40.000) | 0.414 |
| Proportion of MCI | 65 (59.1) | 69 (62.7) | 0.679 |
| Categorical variables are reported as numbers and percentages; continuous variables are reported as means ±SDs. Gender, The gender of the participants; Education, Years of education of the participants; CN, cognitively normal; MCI, mild cognitive impairment; APOE, Apolipoprotein E;Aβ42,Amyloid-β42;T-tau,Total tau; P-tau, Phosphorylated tau; ADAS, Alzheimer's disease assessment scale-cognitive;ADASQ4,ADAS delayed word recall; MMSE, Mini Mental State Examination; RAVLT immediate, Rey Auditory Verbal Learning Test immediate recall; MEM, memory function composite score; EF, executive function composite score. | | | |

**Supplementary Table 6.** Progression risk from CN or MCI to dementia.

|  | Tertile1 |  | Tertile2 |  |  | Tertile3 |  |
| --- | --- | --- | --- | --- | --- | --- | --- |
|  |  |  | HR (95% CI) | P |  | HR (95% CI) | P |
| Baseline clusterin level | Reference |  | 0.832(0.511-1.353) | 0.458 |  | 0.810(0.626-1.047) | 0.810 |
|  |  |  | Reference |  |  | 0.770(0.457-1.296) | 0.325 |

All analyses were corrected for age, gender, educational level, *APOE ε4* status*.*

**Supplementary Table 7.** Interactions effects of clusterin on biomarkers and cognitive measures in MCI participants.

|  | **Age interaction** | | **Sex interaction** | | ***APOE ε4* interaction** | |
| --- | --- | --- | --- | --- | --- | --- |
|  | **β** | **P** | **β** | **P** | **β** | **P** |
| **Baseline** | | | | | | |
| Aβ_42_ | -0.008 | 0.394 | -0.110 | 0.573 | 0.001 | 0.991 |
| T-tau | **0.021** | **0.037** | 0.332 | 0.088 | 0.087 | 0.488 |
| P-tau | 0.020 | 0.052 | 0.370 | 0.062 | 0.099 | 0.437 |
| ADAS11 | 0.013 | 0.267 | 0.423 | 0.066 | 0.050 | 0.731 |
| ADAS13 | 0.009 | 0.416 | 0.292 | 0.200 | 0.081 | 0.569 |
| ADASQ4 | 0.010 | 0.364 | 0.006 | 0.978 | -0.004 | 0.975 |
| MMSE | -0.016 | 0.152 | -0.007 | 0.977 | 0.108 | 0.470 |
| RAVLT immediate | -0.004 | 0.700 | -0.254 | 0.239 | -0.007 | 0.962 |
| MEM | -0.002 | 0.851 | -0.145 | 0.503 | 0.074 | 0.598 |
| EF | **-0.026** | **0.017** | -0.294 | 0.167 | -0.154 | 0.264 |
| Ventricles | 0.011 | 0.173 | 0.127 | 0.416 | -0.074 | 0.466 |
| Hippocampus | **-0.020** | **0.049** | -0.373 | 0.065 | -0.006 | 0.965 |
| Whole brain | **-0.013** | **0.009** | -0.164 | 0.101 | 0.068 | 0.296 |
| Entorhinal | 0.003 | 0.824 | -0.140 | 0.547 | -0.007 | 0.963 |
| Fusiform | -0.003 | 0.783 | -0.006 | 0.975 | 0.093 | 0.468 |
| Mid temporal | **-0.029** | **0.002** | -0.103 | 0.593 | -0.039 | 0.749 |
| **Longitudinal** | | | | | | |
| Aβ_42_ | -0.011 | 0.250 | -0.152 | 0.433 | 0.025 | 0.837 |
| T-tau | 0.003 | 0.757 | 0.089 | 0.689 | 0.182 | 0.199 |
| P-tau | 0.005 | 0.684 | -0.038 | 0.862 | 0.124 | 0.370 |
| ADAS11 | 0.014 | 0.190 | 0.058 | 0.791 | -0.071 | 0.609 |
| ADAS13 | 0.014 | 0.209 | 0.027 | 0.901 | -0.037 | 0.789 |
| ADASQ4 | 0.009 | 0.410 | 0.200 | 0.357 | -0.091 | 0.515 |
| MMSE | -0.006 | 0.555 | -0.176 | 0.446 | 0.125 | 0.389 |
| RAVLT immediate | -0.010 | 0.355 | -0.399 | 0.051 | 0.059 | 0.656 |
| MEM | -0.007 | 0.523 | -0.280 | 0.179 | 0.084 | 0.532 |
| EF | -0.013 | 0.218 | -0.153 | 0.460 | -0.077 | 0.565 |
| Ventricles | 0.014 | 0.153 | 0.136 | 0.465 | 0.104 | 0.391 |
| Hippocampus | -0.003 | 0.768 | -0.114 | 0.572 | 0.008 | 0.953 |
| Whole brain | 0.000 | 0.970 | 0.093 | 0.655 | -0.081 | 0.550 |
| Entorhinal | -0.006 | 0.583 | -0.164 | 0.432 | 0.055 | 0.683 |
| Fusiform | -0.008 | 0.447 | -0.193 | 0.371 | -0.119 | 0.389 |
| Mid temporal | -0.009 | 0.401 | -0.239 | 0.245 | 0.116 | 0.380 |

Significant effects (P <0.05) are shown in bold. All analyses were corrected for age, gender, educational level, *APOEε4* status, and intracranial volume*.*

**Abbreviations**: Aβ42, Amyloid-β42; T-tau, Total tau; P-tau, Phosphorylated tau; ADAS, Alzheimer's disease assessment scale-cognitive; ADASQ4 , ADAS delayed word recall; MMSE, Mini-Mental State Examination; RAVLT immediate, Rey Auditory Verbal Learning Test immediate recall; MEM, memory function composite score; EF, executive function composite score.

**Supplementary Table 8.** Interactions effects of clusterin on biomarkers and cognitive measures in CN participants.

|  | **Age interaction** | | **Sex interaction** | | ***APOE ε4* interaction** | |
| --- | --- | --- | --- | --- | --- | --- |
|  | **β** | **P** | **β** | **P** | **β** | **P** |
| **Baseline** | | | | | | |
| Aβ_42_ | 0.019 | 0.374 | 0.232 | 0.364 | -0.454 | 0.111 |
| T-tau | 0.004 | 0.817 | -0.176 | 0.366 | 0.293 | 0.212 |
| P-tau | 0.005 | 0.761 | -0.205 | 0.303 | 0.439 | 0.067 |
| ADAS11 | 0.000 | 0.986 | -0.230 | 0.300 | 0.234 | 0.381 |
| ADAS13 | 0.006 | 0.746 | -0.306 | 0.148 | 0.112 | 0.663 |
| ADASQ4 | 0.007 | 0.715 | -0.310 | 0.134 | -0.110 | 0.662 |
| MMSE | 0.002 | 0.906 | 0.081 | 0.712 | 0.124 | 0.640 |
| RAVLT immediate | 0.008 | 0.691 | -0.011 | 0.958 | 0.144 | 0.579 |
| MEM | 0.006 | 0.736 | 0.152 | 0.456 | 0.040 | 0.871 |
| EF | 0.012 | 0.528 | -0.019 | 0.928 | -0.277 | 0.271 |
| Ventricles | 0.005 | 0.740 | 0.057 | 0.740 | 0.030 | 0.887 |
| Hippocampus | 0.020 | 0.271 | 0.071 | 0.721 | -0.107 | 0.661 |
| Whole brain | 0.004 | 0.700 | 0.078 | 0.483 | 0.016 | 0.906 |
| Entorhinal | 0.019 | 0.333 | 0.186 | 0.401 | -0.277 | 0.311 |
| Fusiform | -0.023 | 0.098 | 0.112 | 0.465 | 0.235 | 0.214 |
| Mid temporal | -0.002 | 0.903 | 0.096 | 0.501 | **-0.484** | **0.005** |
| **Longitudinal** | | | | | | |
| Aβ_42_ | 0.027 | 0.195 | 0.211 | 0.412 | -0.448 | 0.118 |
| T-tau | -0.008 | 0.701 | -0.180 | 0.434 | 0.363 | 0.191 |
| P-tau | -0.005 | 0.802 | -0.160 | 0.488 | 0.380 | 0.171 |
| ADAS11 | -0.012 | 0.553 | **-0.455** | **0.040** | 0.449 | 0.095 |
| ADAS13 | -0.026 | 0.202 | -0.332 | 0.139 | 0.422 | 0.119 |
| ADASQ4 | -0.012 | 0.545 | -0.174 | 0.438 | 0.208 | 0.446 |
| MMSE | 0.027 | 0.168 | 0.271 | 0.214 | -0.421 | 0.108 |
| RAVLT immediate | 0.027 | 0.167 | 0.341 | 0.120 | -0.321 | 0.230 |
| MEM | 0.034 | 0.084 | 0.364 | 0.098 | -0.468 | 0.079 |
| EF | 0.006 | 0.762 | 0.110 | 0.609 | **-0.603** | **0.019** |
| Ventricles | **-0.045** | **0.024** | 0.035 | 0.877 | **0.629** | **0.019** |
| Hippocampus | 0.022 | 0.255 | 0.174 | 0.430 | **-0.529** | **0.047** |
| Whole brain | 0.009 | 0.679 | 0.164 | 0.478 | -0.433 | 0.120 |
| Entorhinal | **0.047** | **0.010** | 0.240 | 0.253 | **-0.516** | **0.042** |
| Fusiform | 0.006 | 0.761 | 0.305 | 0.170 | **-0.693** | **0.009** |
| Mid temporal | 0.004 | 0.840 | 0.091 | 0.676 | **-0.589** | **0.023** |

Significant effects (P <0.05) are shown in bold. All analyses were corrected for age, gender, educational level, *APOEε4* status, and intracranial volume*.* **Abbreviations**: Aβ42, Amyloid-β42; T-tau, Total tau; P-tau, Phosphorylated tau; ADAS, Alzheimer's disease assessment scale-cognitive; ADASQ4 , ADAS delayed word recall; MMSE, Mini-Mental State Examination; RAVLT immediate, Rey Auditory Verbal Learning Test immediate recall; MEM, memory function composite score; EF, executive function composite score.

**Supplementary Table 9.**Mediation analyses of baseline Clusterin and cognitive measurements with biomarkers as mediators in MCI participants.

|  |  | **a** | **P** | **b** | **P** | **c** | **P** | **c’** | **P** | **Proportion (%)** | **P** |
| --- | --- | --- | --- | --- | --- | --- | --- | --- | --- | --- | --- |
| **Baseline** |  |  |  |  |  |  |  |  |  |  |  |
| Aβ_42_ | EF | 0.225 | 0.007 | 0.227 | 0.034 | **0.191** | **0.035** | 0.128 | 0.196 | 22.68 | 0.166 |
| T-tau |  | **0.44** | **<0.01** | -0.137 | 0.135 | **0.191** | **0.035** | **0.33** | **0.001** | **56.35** | **0.027** |
| P-tau |  | **0.384** | **<0.001** | -0.129 | 0.161 | **0.191** | **0.035** | **0.301** | **0.002** | **42.08** | **0.034** |
| Ventricles |  | **-0.236** | **<0.001** | **-0.391** | **<0.001** | **0.191** | **0.035** | 0.094 | 0.305 | 44.79 | 0.06 |
| Hippocampus |  | **0.195** | **0.02** | **0.324** | **0.007** | **0.191** | **0.035** | 0.194 | 0.053 | 19.9 | 0.057 |
| Fusiform |  | **0.267** | **0.001** | **0.404** | **<0.001** | **0.191** | **0.035** | 0.155 | 0.125 | **36.19** | **0.016** |
| Mid temporal |  | **0.223** | **0.005** | **0.441** | **<0.001** | **0.191** | **0.035** | 0.161 | 0.104 | **33.09** | **0.022** |
| **Longitudinal** |  |  |  |  |  |  |  |  |  |  |  |
| Aβ_42_ | MMSE | **0.243** | **0.003** | **0.389** | **<0.001** | **0.202** | **0.029** | 0.059 | 0.548 | 54 | 0.139 |
|  | RAVLT | **0.243** | **0.003** | **0.346** | **<0.001** | **0.182** | **0.038** | 0.057 | 0.552 | 50.26 | 0.157 |
|  | MEM | **0.243** | **0.003** | **0.327** | **0.002** | **0.186** | **0.036** | 0.06 | 0.531 | 47.09 | 0.167 |
|  | EF | **0.243** | **0.003** | **0.409** | **<0.001** | **0.221** | **0.013** | 0.072 | 0.427 | 52.81 | 0.079 |
| P-tau | MMSE | **-0.227** | **0.013** | 0.136 | 0.142 | **0.202** | **0.029** | **0.252** | **0.008** | 17.84 | 0.079 |
|  | RAVLT | **-0.227** | **0.013** | -0.005 | 0.951 | **0.182** | **0.038** | **0.187** | **0.04** | 3.28 | 0.718 |
|  | MEM | **-0.227** | **0.013** | -0.019 | 0.825 | **0.186** | **0.036** | **0.191** | **0.039** | 2 | 0.797 |
|  | EF | **-0.227** | **0.013** | 0.013 | 0.881 | **0.221** | **0.013** | 0.229 | 0.012 | 5.2 | 0.503 |
| Hippocampus | MMSE | 0.17 | 0.043 | 0.509 | **<0.001** | 0.202 | 0.029 | 0.127 | 0.157 | 44.5 | 0.022 |
|  | RAVLT | 0.17 | 0.043 | 0.363 | **<0.001** | 0.182 | 0.038 | 0.138 | 0.112 | 27.82 | 0.064 |
|  | MEM | 0.17 | 0.043 | 0.38 | **<0.001** | 0.186 | 0.036 | 0.141 | 0.11  4 | 28.02 | 0.067 |
|  | EF | **0.17** | **0.043** | **0.312** | **0.002** | **0.221** | **0.013** | **0.198** | **0.02** | **17.87** | **0.045** |
| Whole brain | MMSE | 0.177 | 0.047 | 0.253 | 0.008 | 0.202 | 0.029 | 0.158 | 0.092 | 21.41 | 0.065 |
|  | RAVLT | 0.177 | 0.047 | 0.202 | 0.019 | 0.182 | 0.038 | 0.138 | 0.11 | 16.56 | 0.12 |
|  | MEM | 0.177 | 0.047 | 0.214 | 0.015 | 0.186 | 0.036 | 0.148 | 0.096 | 16.85 | 0.11 |
|  | EF | **0.177** | **0.047** | **0.221** | **0.012** | **0.221** | **0.013** | **0.179** | **0.042** | 15.33 | 0.06 |
| Mid temporal | MMSE | **0.177** | **0.04** | 0.516 | **<0.001** | **0.202** | **0.029** | 0.145 | 0.103 | **36.94** | **0.045** |
|  | RAVLT | **0.177** | **0.04** | 0.447 | **<0.001** | **0.182** | **0.038** | 0.12 | 0.152 | 36.79 | 0.058 |
|  | MEM | 0.177 | **0.04** | 0.512 | **<0.001** | 0.186 | 0.036 | 0.114 | 0.174 | 41.71 | 0.06 |
|  | EF | **0.177** | **0.04** | **0.5** | **<0.001** | **0.221** | **0.013** | 0.162 | 0.052 | **33.21** | **0.045** |

Significant effects (P <0.05) are shown in bold. All analyses were corrected for age, gender, educational level, *APOEε4* status, and intracranial volume*.* **Abbreviations**: Aβ_42_, Amyloid-β_42_; T-tau, Total tau; P-tau, Phosphorylated tau; MMSE, Mini-Mental State Examination; RAVLT, Rey Auditory Verbal Learning Test immediate recall; MEM, memory function composite score; EF, executive function composite score.

**Supplementary Table 10.**Mediation analyses of baseline Clusterin and cognitive measurements with biomarkers as mediators in MCI participants.

|  |  | **a** | **P** | **b** | **P** | **c** | **P** | **c’** | **P** | **Proportion (%)** | **P** |  |
| --- | --- | --- | --- | --- | --- | --- | --- | --- | --- | --- | --- | --- |
| **Baseline** |  |  |  |  |  |  |  |  |  |  |  |  |
| Aβ42 | Ventricles | 0.225 | 0.007 | -0.108 | 0.163 | -0.236 | **<0.001** | -0.236 | **<0.001** | 5.920 | 0.477 |  |
|  | Hippocampus | 0.225 | 0.007 | 0.148 | 0.124 | 0.195 | **0.020** | 0.195 | **0.020** | 16.890 | 0.340 |  |
|  | Whole brain | 0.225 | 0.007 | 0.034 | 0.499 | 0.048 | 0.257 | 0.048 | 0.257 | 6.100 | 0.810 |  |
|  | Entorhinal | 0.225 | 0.007 | 0.217 | **0.043** | 0.157 | 0.096 | 0.157 | 0.096 | 32.010 | 0.324 |  |
|  | Fusiform | 0.225 | 0.007 | 0.134 | 0.175 | 0.267 | **0.002** | 0.267 | **0.002** | 6.400 | 0.514 |  |
|  | Mid temporal | 0.225 | 0.007 | 0.055 | 0.547 | 0.223 | **0.005** | 0.223 | **0.005** | 0.100 | 0.987 |  |
| T-tau | Ventricles | 0.440 | **<0.001** | -0.224 | **0.001** | -0.236 | **<0.001** | -0.236 | **<0.001** | 46.270 | **0.012** |  |
|  | Hippocampus | 0.440 | **<0.001** | 0.101 | 0.259 | 0.195 | **0.020** | 0.195 | **0.020** | 3.500 | 0.857 |  |
|  | Whole brain | 0.440 | **<0.001** | -0.017 | 0.692 | 0.048 | 0.257 | 0.048 | 0.257 | 3.560 | 0.857 |  |
|  | Entorhinal | 0.440 | **<0.001** | 0.064 | 0.513 | 0.157 | 0.096 | 0.157 | 0.096 | 23.150 | 0.560 |  |
|  | Fusiform | 0.440 | **<0.001** | 0.114 | 0.207 | 0.267 | **0.002** | 0.267 | **0.002** | 2.600 | 0.841 |  |
|  | Mid temporal | 0.440 | **<0.001** | 0.006 | 0.946 | 0.223 | **0.005** | 0.223 | **0.005** | 20.960 | 0.204 |  |
| P-tau | Ventricles | 0.384 | **<0.001** | -0.216 | **0.002** | -0.236 | **<0.001** | -0.236 | **<0.001** | 20.520 | **0.049** |  |
|  |  |  |  |  |  |  |  |  |  |  |  |  |
|  | Hippocampus | 0.384 | **<0.001** | 0.136 | 0.128 | 0.195 | **0.020** | 0.195 | **0.020** | 12.830 | 0.459 |  |
|  | Whole brain | 0.384 | **<0.001** | -0.014 | 0.757 | 0.048 | 0.257 | 0.048 | 0.257 | 15.110 | 0.630 |  |
|  | Entorhinal | 0.384 | **<0.001** | 0.090 | 0.360 | 0.157 | 0.096 | 0.157 | 0.096 | 0.180 | 0.986 |  |
|  | Fusiform | 0.384 | **<0.001** | 0.113 | 0.209 | 0.267 | **0.002** | 0.267 | **0.002** | 0.242 | 0.982 |  |
|  | Mid temporal | 0.384 | **<0.001** | 0.015 | 0.859 | 0.223 | **0.005** | 0.223 | **0.005** | 13.160 | 0.349 |  |
| Longitudinal |  |  |  |  |  |  |  |  |  |  |  |  |
| Aβ42 | Ventricles | 0.243 | **0.003** | -0.324 | **0.000** | -0.106 | 0.180 | 0.066 | 0.394 | 60.080 | 0.842 |  |
|  | Hippocampus | 0.243 | **0.003** | 0.326 | **0.001** | 0.170 | **0.043** | 0.032 | 0.716 | 59.050 | 0.202 |  |
|  | Whole brain | 0.243 | **0.003** | 0.284 | **0.006** | 0.177 | **0.047** | 0.063 | 0.511 | 42.250 | 0.189 |  |
|  | Entorhinal | 0.243 | **0.003** | 0.287 | **0.005** | 0.131 | 0.132 | 0.034 | 0.722 | 49.710 | 0.281 |  |
|  | Fusiform | 0.243 | **0.003** | 0.206 | **0.042** | 0.114 | 0.201 | -0.038 | 0.692 | 25.788 | 0.844 |  |
|  | Mid temporal | 0.243 | **0.003** | 0.302 | **0.003** | 0.177 | **0.039** | 0.045 | 0.631 | 49.610 | 0.209 |  |
| T-tau | Ventricles | -0.095 | 0.307 | -0.016 | 0.835 | -0.106 | 0.180 | -0.118 | 0.142 | 0.656 | 0.850 |  |
|  | Hippocampus | -0.095 | 0.307 | 0.042 | 0.601 | 0.170 | **0.043** | 0.176 | **0.036** | 1.401 | 0.684 |  |
|  | Whole brain | -0.095 | 0.307 | -0.023 | 0.791 | 0.177 | **0.047** | 0.177 | **0.050** | 0.114 | 0.949 |  |
|  | Entorhinal | -0.095 | 0.307 | 0.100 | 0.232 | 0.131 | 0.132 | 0.147 | 0.096 | 4.450 | 0.524 |  |
|  | Fusiform | -0.095 | 0.307 | -0.032 | 0.712 | 0.114 | 0.201 | 0.106 | 0.246 | 0.552 | 0.880 |  |
|  | Mid temporal | -0.095 | 0.307 | 0.004 | 0.959 | 0.177 | **0.039** | 0.174 | **0.047** | 0.314 | 0.885 |  |
| P-tau | Ventricles | -0.227 | **0.013** | -0.166 | **0.029** | -0.106 | 0.180 | -0.160 | **0.045** | 31.187 | 0.178 |  |
|  |  |  |  |  |  |  |  |  |  |  |  |  |
|  | Hippocampus | -0.227 | **0.013** | 0.180 | **0.026** | 0.170 | **0.043** | 0.219 | **0.009** | 31.678 | 0.176 |  |
|  | Whole brain | -0.227 | **0.013** | 0.060 | 0.492 | 0.177 | **0.047** | 0.200 | **0.029** | 9.552 | 0.318 |  |
|  | Entorhinal | -0.227 | **0.013** | 0.136 | 0.110 | 0.131 | 0.132 | 0.173 | 0.052 | 15.550 | 0.380 |  |
|  | Fusiform | -0.227 | **0.013** | 0.094 | 0.282 | 0.114 | 0.201 | 0.134 | 0.147 | 15.610 | 0.390 |  |
|  | Mid temporal | -0.227 | **0.013** | 0.116 | 0.169 | 0.177 | **0.039** | 0.206 | **0.019** | 17.469 | 0.123 |  |

Significant effects (P <0.05) are shown in bold. All analyses were corrected for age, gender, educational level, *APOEε4* status, and intracranial volume*.* **Abbreviations**: Aβ_42_, Amyloid-β_42_; T-tau, Total tau; P-tau, Phosphorylated tau; MMSE, Mini-Mental State Examination; RAVLT, Rey Auditory Verbal Learning Test immediate recall; MEM, memory function composite score; EF, executive function composite score.

**Supplementary Table 11**Mediation analyses of baseline Clusterin and cognitive measurements with biomarkers as mediators in CN participants.

|  |  | **a** | **P** | **b** | **P** | **c** | **P** | **c’** | **P** | **Proportion (%)** | **P** |  |
| --- | --- | --- | --- | --- | --- | --- | --- | --- | --- | --- | --- | --- |
| **Baseline** |  |  |  |  |  |  |  |  |  |  |  |  |
| Aβ42 | Ventricles | 0.248 | 0.079 | -0.131 | 0.193 | -0.073 | 0.409 | 0.026 | 0.819 | 2.590 | 0.930 |  |
|  | Hippocampus | 0.248 | 0.079 | 0.087 | 0.477 | -0.170 | 0.096 | -0.136 | 0.312 | 6.690 | 0.690 |  |
|  | Whole brain | 0.248 | 0.079 | -0.004 | 0.955 | -0.008 | 0.888 | -0.052 | 0.512 | 0.902 | 0.990 |  |
|  | Entorhinal | 0.248 | 0.079 | 0.018 | 0.895 | -0.002 | 0.983 | 0.020 | 0.900 | 0.748 | 0.960 |  |
|  | Fusiform | 0.248 | 0.079 | 0.001 | 0.992 | -0.050 | 0.528 | 0.023 | 0.825 | 0.245 | 0.980 |  |
|  | Mid temporal | 0.248 | 0.079 | 0.107 | 0.234 | 0.111 | 0.131 | 0.003 | 0.976 | 6.959 | 0.820 |  |
| T-tau | Ventricles | 0.426 | **<0.001** | -0.143 | 0.086 | -0.073 | 0.409 | -0.005 | 0.955 | 3.154 | 0.920 |  |
|  | Hippocampus | 0.426 | **<0.001** | -0.085 | 0.421 | -0.170 | 0.096 | -0.170 | 0.131 | 0.016 | 1.000 |  |
|  | Whole brain | 0.426 | **<0.001** | 0.056 | 0.323 | -0.008 | 0.888 | -0.043 | 0.490 | 13.060 | 0.650 |  |
|  | Entorhinal | 0.426 | **<0.001** | 0.031 | 0.795 | -0.002 | 0.983 | -0.016 | 0.903 | 1.070 | 0.940 |  |
|  | Fusiform | 0.426 | **<0.001** | -0.062 | 0.446 | -0.050 | 0.528 | -0.028 | 0.749 | 2.200 | 0.900 |  |
|  | Mid temporal | 0.426 | **<0.001** | 0.088 | 0.243 | 0.111 | 0.131 | 0.086 | 0.282 | 0.587 | 0.960 |  |
| P-tau | Ventricles | 0.371 | **<0.001** | -0.124 | 0.134 | -0.073 | 0.409 | -0.022 | 0.804 | 2.645 | 0.910 |  |
|  |  |  |  |  |  |  |  |  |  |  |  |  |
|  | Hippocampus | 0.371 | **<0.001** | -0.073 | 0.493 | -0.170 | 0.096 | -0.172 | 0.117 | 0.138 | 0.980 |  |
|  | Whole brain | 0.371 | **<0.001** | 0.065 | 0.247 | -0.008 | 0.888 | -0.041 | 0.499 | 9.680 | 0.700 |  |
|  | Entorhinal | 0.371 | **<0.001** | -0.004 | 0.974 | -0.002 | 0.983 | 0.002 | 0.988 | 0.354 | 0.960 |  |
|  | Fusiform | 0.371 | **<0.001** | -0.022 | 0.792 | -0.050 | 0.528 | -0.048 | 0.577 | 1.842 | 0.880 |  |
|  | Mid temporal | 0.371 | **<0.001** | 0.064 | 0.398 | 0.111 | 0.131 | 0.098 | 0.212 | 0.041 | 0.990 |  |
| Longitudinal |  |  |  |  |  |  |  |  |  |  |  |  |
| Aβ42 | Ventricles | 0.240 | 0.092 | -0.332 | **0.014** | 0.035 | 0.767 | 0.077 | 0.604 | 58.710 | 0.863 |  |
|  | Hippocampus | 0.240 | 0.092 | 0.428 | **0.002** | -0.123 | 0.297 | -0.311 | **0.032** | 58.020 | 0.201 |  |
|  | Whole brain | 0.240 | 0.092 | 0.106 | 0.477 | -0.122 | 0.320 | -0.118 | 0.482 | 40.925 | 0.198 |  |
|  | Entorhinal | 0.240 | 0.092 | 0.267 | **0.043** | -0.051 | 0.650 | -0.117 | 0.425 | 50.710 | 0.272 |  |
|  | Fusiform | 0.240 | 0.092 | 0.293 | **0.043** | -0.016 | 0.889 | -0.129 | 0.422 | 25.386 | 0.845 |  |
|  | Mid temporal | 0.240 | 0.092 | 0.486 | **0.000** | 0.097 | 0.399 | 0.024 | 0.865 | 49.890 | 0.214 |  |
| T-tau | Ventricles | 0.028 | 0.816 | 0.047 | 0.672 | 0.035 | 0.767 | 0.038 | 0.751 | 0.609 | 0.850 |  |
|  | Hippocampus | 0.028 | 0.816 | 0.061 | 0.581 | -0.123 | 0.297 | -0.124 | 0.297 | 1.501 | 0.665 |  |
|  | Whole brain | 0.028 | 0.816 | -0.145 | 0.203 | -0.122 | 0.320 | -0.114 | 0.348 | 0.191 | 0.925 |  |
|  | Entorhinal | 0.028 | 0.816 | 0.141 | 0.175 | -0.051 | 0.650 | -0.056 | 0.618 | 4.180 | 0.550 |  |
|  | Fusiform | 0.028 | 0.816 | -0.038 | 0.730 | -0.016 | 0.889 | -0.017 | 0.885 | 0.590 | 0.880 |  |
|  | Mid temporal | 0.028 | 0.816 | -0.024 | 0.826 | 0.097 | 0.399 | 0.095 | 0.412 | 0.317 | 0.887 |  |
| P-tau | Ventricles | 0.014 | 0.908 | -0.092 | 0.403 | 0.035 | 0.767 | 0.040 | 0.737 | 32.048 | 0.165 |  |
|  |  |  |  |  |  |  |  |  |  |  |  |  |
|  | Hippocampus | 0.014 | 0.908 | 0.110 | 0.316 | -0.123 | 0.297 | -0.124 | 0.295 | 26.484 | 0.063 |  |
|  | Whole brain | 0.014 | 0.908 | -0.104 | 0.360 | -0.122 | 0.320 | -0.116 | 0.341 | 10.797 | 0.313 |  |
|  | Entorhinal | 0.014 | 0.908 | 0.199 | 0.054 | -0.051 | 0.650 | -0.055 | 0.621 | 21.620 | 0.194 |  |
|  | Fusiform | 0.014 | 0.908 | 0.093 | 0.399 | -0.016 | 0.889 | -0.019 | 0.872 | 15.570 | 0.370 |  |
|  | Mid temporal | 0.014 | 0.908 | 0.094 | 0.383 | 0.097 | 0.399 | 0.094 | 0.418 | 17.590 | 0.126 |  |

Significant effects (P <0.05) are shown in bold. All analyses were corrected for age, gender, educational level, *APOEε4* status, and intracranial volume*.* **Abbreviations**: Aβ_42_, Amyloid-β_42_; T-tau, Total tau; P-tau, Phosphorylated tau; MMSE, Mini-Mental State Examination; RAVLT, Rey Auditory Verbal Learning Test immediate recall; MEM, memory function composite score; EF, executive function composite score.

**Supplementary Table 12.** main effects of clusterin on biomarkers and cognitive measures in MCI participants.

| Age | ≤65 | | ***＞65*** | |
| --- | --- | --- | --- | --- |
|  | **β** | **P** | **β** | **P** |
| **Baseline** |  |  |  |  |
| Aβ_42_ | **0.713** | **0.025** | 0.146 | 0.098 |
| T-tau | 0.191 | 0.438 | **0.536** | **<0.001** |
| P-tau | 0.124 | 0.618 | **0.483** | **<0.001** |
| ADAS11 | -0.177 | 0.354 | -0.139 | 0.185 |
| ADAS13 | -0.036 | 0.814 | -0.110 | 0.290 |
| ADASQ4 | 0.144 | 0.504 | 0.017 | 0.863 |
| MMSE | 0.638 | 0.107 | 0.119 | 0.248 |
| RAVLT immediate | 0.339 | 0.127 | -0.002 | 0.987 |
| MEM | 0.114 | 0.584 | 0.062 | 0.533 |
| EF | 0.298 | 0.226 | 0.110 | 0.264 |
| Ventricles | -0.171 | 0.714 | **-0.219** | **0.002** |
| Hippocampus | 0.835 | 0.101 | 0.126 | 0.180 |
| Whole brain | 0.122 | 0.703 | 0.033 | 0.449 |
| Entorhinal | 0.018 | 0.945 | 0.120 | 0.258 |
| Fusiform | 0.632 | 0.092 | **0.247** | **0.009** |
| Mid temporal | 0.578 | 0.090 | 0.126 | 0.138 |
| **Longitudinal** |  |  |  |  |
| Aβ_42_ | **0.656** | **0.029** | 0.172 | 0.051 |
| T-tau | -0.376 | 0.240 | -0.055 | 0.585 |
| P-tau | -0.437 | 0.192 | **-0.225** | **0.024** |
| ADAS11 | -0.237 | 0.296 | -0.144 | 0.144 |
| ADAS13 | -0.244 | 0.323 | -0.075 | 0.444 |
| ADASQ4 | -0.233 | 0.498 | -0.057 | 0.569 |
| MMSE | **0.524** | **0.006** | 0.145 | 0.151 |
| RAVLT immediate | **0.696** | **0.006** | 0.133 | 0.159 |
| MEM | **0.565** | **0.009** | 0.142 | 0.142 |
| EF | **0.616** | **0.016** | 0.143 | 0.133 |
| Ventricles | -0.318 | 0.324 | 0.005 | 0.954 |
| Hippocampus | 0.262 | 0.520 | 0.117 | 0.199 |
| Whole brain | -0.060 | 0.910 | 0.157 | 0.101 |
| Entorhinal | **0.432** | **0.420** | 0.093 | 0.325 |
| Fusiform | 0.446 | 0.292 | 0.021 | 0.832 |
| Mid temporal | 0.147 | 0.666 | 0.130 | 0.164 |

Significant effects (P <0.05) are shown in bold. All analyses were corrected for age, gender, educational level, *APOEε4* status, and intracranial volume*.* **Abbreviations**: Aβ42, Amyloid-β42; T-tau, Total tau; P-tau, Phosphorylated tau; ADAS, Alzheimer's disease assessment scale-cognitive; ADASQ4 , ADAS delayed word recall; MMSE, Mini-Mental State Examination; RAVLT immediate, Rey Auditory Verbal Learning Test immediate recall; MEM, memory function composite score; EF, executive function composite score.

**Supplementary Table 13 .** main effects of clusterin on biomarkers and cognitive measures in MCI participants.

| Gender | Female | | Male | |
| --- | --- | --- | --- | --- |
| **Baseline** | **β** | **P** | **β** | **P** |
| Aβ_42_ | 0.276 | 0.123 | **0.184** | **0.044** |
| T-tau | 0.197 | 0.235 | **0.497** | **<0.001** |
| P-tau | 0.135 | 0.423 | **0.452** | **<0.001** |
| ADAS11 | -0.338 | 0.054 | -0.119 | 0.288 |
| ADAS13 | -0.276 | 0.110 | -0.115 | 0.304 |
| ADASQ4 | -0.022 | 0.888 | -0.038 | 0.731 |
| MMSE | 0.132 | 0.493 | 0.183 | 0.098 |
| RAVLT immediate | 0.239 | 0.136 | -0.027 | 0.809 |
| MEM | 0.193 | 0.224 | 0.048 | 0.663 |
| EF | **0.337** | **0.039** | 0.134 | 0.217 |
| Ventricles | **-0.273** | **0.043** | **-0.238** | **0.005** |
| Hippocampus | **0.422** | **0.013** | 0.125 | 0.201 |
| Whole brain | 0.158 | 0.105 | 0.024 | 0.673 |
| Entorhinal | 0.281 | 0.100 | 0.113 | 0.346 |
| Fusiform | 0.251 | 0.157 | **0.292** | **0.007** |
| Mid temporal | 0.279 | 0.090 | **0.238** | **0.029** |
| **Longitudinal** |  |  |  |  |
| Aβ_42_ | **0.344** | **0.049** | **0.199** | **0.033** |
| T-tau | -0.158 | 0.340 | -0.062 | 0.568 |
| P-tau | -0.157 | 0.326 | **-0.221** | **0.043** |
| ADAS11 | -0.217 | 0.201 | -0.166 | 0.132 |
| ADAS13 | -0.197 | 0.225 | -0.105 | 0.336 |
| ADASQ4 | **-0.367** | **0.012** | -0.036 | 0.744 |
| MMSE | 0.279 | 0.142 | 0.192 | 0.082 |
| RAVLT immediate | **0.459** | **0.002** | 0.094 | 0.380 |
| MEM | **0.416** | **0.005** | 0.124 | 0.254 |
| EF | **0.329** | **0.042** | 0.184 | 0.086 |
| Ventricles | -0.170 | 0.242 | -0.090 | 0.363 |
| Hippocampus | 0.226 | 0.155 | 0.154 | 0.133 |
| Whole brain | 0.109 | 0.502 | 0.181 | 0.084 |
| Entorhinal | 0.217 | 0.183 | 0.092 | 0.385 |
| Fusiform | 0.243 | 0.156 | 0.073 | 0.510 |
| Mid temporal | 0.298 | 0.064 | 0.134 | 0.208 |

Significant effects (P <0.05) are shown in bold. All analyses were corrected for age, gender, educational level, *APOEε4* status, and intracranial volume*.* **Abbreviations**: Aβ42, Amyloid-β42; T-tau, Total tau; P-tau, Phosphorylated tau; ADAS, Alzheimer's disease assessment scale-cognitive; ADASQ4 , ADAS delayed word recall; MMSE, Mini-Mental State Examination; RAVLT immediate, Rey Auditory Verbal Learning Test immediate recall; MEM, memory function composite score; EF, executive function composite score.

**Supplementary Table 14 .** main effects of clusterin on biomarkers and cognitive measures in MCI participants.

| Aggregated Aβ | A+ | | A- | |
| --- | --- | --- | --- | --- |
| **Baseline** | **β** | **P** | **β** | **P** |
| Aβ_42_ | 0.167 | 0.090 | 0.094 | 0.727 |
| T-tau | **0.558** | **<0.001** | **0.470** | **0.003** |
| P-tau | **0.532** | **<0.001** | **0.446** | **0.007** |
| ADAS11 | -0.031 | 0.789 | -0.271 | 0.127 |
| ADAS13 | 0.020 | 0.863 | -0.222 | 0.242 |
| ADASQ4 | 0.119 | 0.270 | -0.091 | 0.661 |
| MMSE | 0.123 | 0.304 | 0.092 | 0.640 |
| RAVLT immediate | -0.059 | 0.589 | 0.106 | 0.551 |
| MEM | -0.028 | 0.802 | 0.109 | 0.565 |
| EF | 0.108 | 0.308 | 0.047 | 0.816 |
| Ventricles | **-0.229** | **0.005** | -0.096 | 0.484 |
| Hippocampus | 0.120 | 0.270 | 0.171 | 0.290 |
| Whole brain | 0.058 | 0.226 | -0.167 | 0.139 |
| Entorhinal | 0.047 | 0.712 | 0.126 | 0.481 |
| Fusiform | **0.299** | **0.007** | 0.042 | 0.800 |
| Mid temporal | **0.192** | **0.048** | 0.071 | 0.719 |
| **Longitudinal** |  |  |  |  |
| Aβ_42_ | **0.228** | **0.021** | -0.121 | 0.599 |
| T-tau | -0.089 | 0.425 | -0.039 | 0.851 |
| P-tau | **-0.304** | **0.005** | 0.007 | 0.972 |
| ADAS11 | -0.026 | 0.813 | -0.280 | 0.170 |
| ADAS13 | 0.009 | 0.933 | -0.272 | 0.173 |
| ADASQ4 | -0.083 | 0.452 | -0.189 | 0.344 |
| MMSE | 0.096 | 0.424 | 0.238 | 0.201 |
| RAVLT immediate | 0.078 | 0.467 | 0.212 | 0.310 |
| MEM | 0.089 | 0.411 | 0.241 | 0.234 |
| EF | 0.099 | 0.354 | 0.159 | 0.427 |
| Ventricles | 0.128 | 0.138 | -0.342 | 0.071 |
| Hippocampus | 0.003 | 0.974 | 0.301 | 0.127 |
| Whole brain | 0.046 | 0.666 | **0.476** | **0.019** |
| Entorhinal | 0.002 | 0.982 | 0.170 | 0.321 |
| Fusiform | -0.092 | 0.392 | 0.222 | 0.216 |
| Mid temporal | 0.095 | 0.370 | 0.083 | 0.680 |

Significant effects (P <0.05) are shown in bold. All analyses were corrected for age, gender, educational level, *APOEε4* status, and intracranial volume*.* **Abbreviations**: Aβ42, Amyloid-β42; T-tau, Total tau; P-tau, Phosphorylated tau; ADAS, Alzheimer's disease assessment scale-cognitive; ADASQ4 , ADAS delayed word recall; MMSE, Mini-Mental State Examination; RAVLT immediate, Rey Auditory Verbal Learning Test immediate recall; MEM, memory function composite score; EF, executive function composite score.
